# 5-Benzyliden-2-(5-methylthiazol-2-ylimino)thiazolidin-4-ones as Antimicrobial Agents. Design, Synthesis, Biological Evaluation and Molecular Docking Studies

**DOI:** 10.3390/antibiotics10030309

**Published:** 2021-03-17

**Authors:** Michelyne Haroun, Christophe Tratrat, Aggeliki Kolokotroni, Anthi Petrou, Athina Geronikaki, Marija Ivanov, Marina Kostic, Marina Sokovic, Alejandro Carazo, Přemysl Mladěnka, Nagaraja Sreeharsha, Katharigatta N. Venugopala, Anroop B. Nair, Heba S. Elsewedy

**Affiliations:** 1 Department of Pharmaceutical Sciences, College of Clinical Pharmacy, King Faisal University, Al-Ahsa 31982, Saudi Arabia; christophetratrat@gmail.com (C.T.); sharsha@kfu.edu.sa (N.S.); kvenugopala@kfu.edu.sa (K.N.V.); anair@kfu.edu.sa (A.B.N.); helsewedy@kfu.edu.sa (H.S.E.); 2 School of Health, Department of Pharmacy, Aristotle University of Thessaloniki, 54124 Thessaloniki, Greece; aggelikikolokotroni@gmail.com (A.K.); anthi.petrou.thessaloniki1@gmail.com (A.P.); 3 Mycological Laboratory, Department of Plant Physiology, Institute for Biological Research, Siniša Stanković-National Institute of Republic of Serbia, University of Belgrade, Bulevar Despota Stefana 142, 11000 Belgrade, Serbia; marija.smiljkovic@ibiss.bg.ac.rs (M.I.); marina.kostic@ibiss.bg.ac.rs (M.K.); mris@ibiss.bg.ac.rs (M.S.); 4 Department of pharmacology and toxicology, Faculty of Pharmacy, Charles University, 500 05 Hradec Králové, Czech Republic; carazofa@faf.cuni.cz (A.C.); mladenkap@faf.cuni.cz (P.M.); 5 Department of Biotechnology and Food Technology, Faculty of Applied Sciences, Durban University of Technology, Durban 4001, South Africa

**Keywords:** thiazolidinones, antibacterial, antifungal, microdilution method, docking, MurB, CYP51

## Abstract

In this study, we report the design, synthesis, computational and experimental evaluation of the antimicrobial activity, as well as docking studies of new 5-methylthiazole based thiazolidinones. All compounds demonstrated antibacterial efficacy, some of which (**1**, **4**, **10** and **13)** exhibited good activity against *E. coli* and *B. cereus.* The evaluation of antibacterial activity against three resistant strains, MRSA, *P. aeruginosa and E. coli*, revealed that compound **12** showed the best activity, higher than reference drugs ampicillin and streptomycin, which were inactive or exhibited only bacteriostatic activity against MRSA, respectively. Ten out of fifteen compounds demonstrated higher potency than reference drugs against a resistant strain of *E. coli,* which appeared to be the most sensitive species to our compounds. Compounds **8**, **13** and **14** applied in a concentration equal to MIC reduced *P. aeruginosa* biofilm formation by more than 50%. All compounds displayed antifungal activity, with compound **10** being the most active. The majority of compounds showed better activity than ketoconazole against almost all fungal strains. In order to elucidate the mechanism of antibacterial and antifungal activities, molecular docking studies on *E. coli* Mur B and *C. albicans* CYP51 and dihydrofolate reductase were performed. Docking analysis of *E. coli* MurB indicated a probable involvement of MurB inhibition in the antibacterial mechanism of tested compounds while docking to 14α-lanosterol demethylase (CYP51) and tetrahydrofolate reductase of *Candida albicans* suggested that probable involvement of inhibition of CYP51 reductase in the antifungal activity of the compounds. Potential toxicity toward human cells is also reported.

## 1. Introduction

Despite the achievements in the treatment of infective diseases during the last 50 years, unfortunately, the new infections affecting large populations are instigating significant morbidity and mortality. The most recent case of COVID-19 is the best example. Another crucial problem is the growth of antibiotic resistance (AMR), which represents a significant obstacle for the successful treatment of severe and particularly life-treating infections. The phenomenon of AMR at biochemical and physiological levels may manifest in any single bacterial cell (planktonic growth) or in a sessile complex microbial community (biofilm) [1,2].

One of the bacterial growth modes is the development of biofilms, which may be considered a basic endurance strategy in hostile environments [2]. Biofilm formation plays an essential role in bacterial infection and antimicrobial resistance. In general, biofilm-embedded bacteria are more resistant to common antimicrobial agents and host defense systems than bacteria in the planktonic state [3]. Established biofilms are less sensitive to elimination by the immune system, and this leads to chronic and persistent infections [4]. The major pathogen, which causes biofilm-associated infections, is *Pseudomonas aeruginosa.* It is able to form biofilm on a broad range of surfaces [5,6]. Similarly, there are reports in the literature that biofilm of *Candida* may be up to 1000 times more resistant to antifungal agents compared to their planktonic cell [7].

The increasing resistance to the current antimicrobial treatment has resulted in a crucial need for the discovery and development of novel entities for the treatment of infections with different modes of action that could target both sensitive and, in particular resistant strains [8]. This need is even greater for patients suffering from chronic inflammatory bowel diseases as well as cystic fibrosis (CF). During an inflammatory response in the gut, some commensal microorganisms, such as *E. coli* and *C. albicans,* can thrive and contribute to illness [9]. While *Pseudomonas aeruginosa* is an opportunistic pathogen that commonly infects the CF lung, promoting an accelerated decline of pulmonary function [10].

Although new drugs were introduced in the battle against bacteria and fungi, such as echinocandin derivatives [11,12], nevertheless, some fungal species are still resistant.

Consequently, the major clinical challenge is to overcome the need for further new antimicrobial agents that can simultaneously combat the resistance dilemma by designing powerful new drugs being less prone to multiresistance or to toxic side effects.

Thiazoles have been found to hold a wide array of pharmacological actions with importance in biological systems. Among the activities mentioned are antimicrobial [13,14,15,16,17], local anesthetic [18], anti-inflammatory [19,20,21], anti-HIV [22,23], analgesic and antipyretic [24], antidiabetic [25,26], anticancer [27,28], antioxidant [29] and many others [30,31]. On the other hand, thiazolidinone core attracted the interest of scientists due to its multiple degrees of medicinal and biological activities [20,23,32,33,34,35,36,37,38]. Antifungal and antibacterial activities of various thiazole and thiazolidinone skeletons have been extensively studied [20,39,40,41,42].

Herein, we illustrate the molecular docking design, preparation and assessment of antimicrobial potency of fifteen new 5-benzyliden-2-(5-methylthiazol-2-ylimino) thiazolidin-4-ones. The design of many thiazolidinones has been accomplished by entering various arylidene groups at the 5 position of the thiazolidinone cores, since as reported in our earlier investigations [43], which were recently exploited as bioactive arms on heterocycle scaffolds useful to encompass certain physicochemical properties as hydrophobic and steric.

## 2. Results and Discussion

### 2.1. Toxicity Prediction

Compound toxicity was predicted utilizing the OpenTox and CBLIGAND programs designed according to the REACH legislation requirements, which favors the practice of alternative testing manipulations to diminish animal experiment practices in testing toxicity. These programs, with the help of in silico standards and algorithms, perform an assessment of the cytotoxicity of compounds [44,45].

The results of the toxicity prediction in rats and mice suggested a lack of carcinogenicity and mutagenesis as well as no toxicity to the skin and eyes (Table S1). However, these remain predictive data that cannot assure the safety of these compounds**.**

### 2.2. Chemistry

**Scheme 1:**
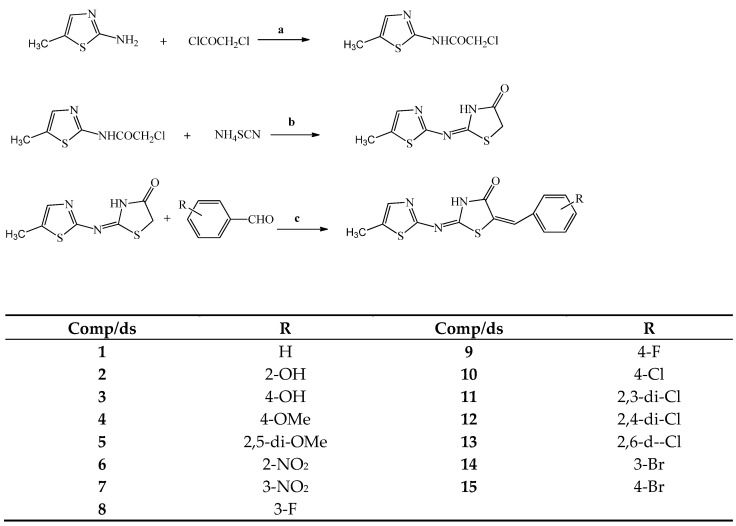
Synthesis of titled compounds.

The designed compounds were synthesized according to the process described in our previous papers [20,43] and are shown in Scheme 1.

Compounds **1**–**15** exist as potential E and Z geometrical isomers; the Z conformation of the 5 exocyclic C=C double bonds was assigned on the basis of ^1^HNMR spectroscopy and on the basis of literature data for analogous 4-thiazolidinones and 2,4-thiazolidinones [43,46,47]. The ^1^H NMR spectra of compounds **1**–**15** showed only one kind of methine proton that, deshielded by the adjacent C=O. It was detected at 7.63–7.97 ppm at higher chemical shift values than the expected ones for E isomers that have a methine proton with a lesser deshielding effect. All compounds were characterized spectroscopically (IR, ^1^H-NMR, C-NMR, MS).

### 2.3. Biological Evaluation

#### 2.3.1. Antibacterial Action

The derivatives were evaluated for their inhibitory action on the growth of eight bacterial strains in addition to eight fungi using the microdilution method with the aim of disclosing minimal inhibitory (MIC), minimal bactericidal (MBC) and minimal fungicidal (MFC) concentrations. All compounds demonstrated antibacterial efficiency, but their potency was different. MIC were in the range of 26.3–378.5 μM and MBCs ranged between 52.6–757.0 μM (Table 1). The antibacterial activity of these compounds can be presented in the following order: **7** > **2** > **14** > **8** > **STM** > **13** > **4** > **6** > **3** > **10** > **11** > **5** > **12** > **1** > **15** > **9** > **AMP**. Compound **7** exhibited the highest antibacterial activity. Its bacteriostatic activity was detected in concentrations of 43.3–86.7 µM and its bactericidal activity at concentrations of 86.7–173.4 μM. This compound is 6 times more potent than ampicillin and 3 times than streptomycin against all bacteria. Compound **9** was the less active (MIC ranging from 125.4 to 344.8 μM and MBC ranging from 250.7 to 689.6 μM). Surprisingly, most of the derivatives were more efficient than ampicillin and compounds **2**–**8**, **10**, **11, 13** and **14** exhibited higher potency than streptomycin towards the majority of tested bacterial pathogens.

Furthermore, these derivatives exhibited superior potency compared to the references (streptomycin and ampicillin) against *E. coli* and *L. monocytogenes*. The same good activity was observed against *S. typhimurium* and *S. aureus*.

Thus, compounds **1**, **4**, **10** and **13** exhibited good activity against *E. coli* and *B. cereus* with MIC ranging from 26.3 to 40.5 µM and MBC ranging from 52.6 to 81.0 µM., whereas derivatives **7** and **8** were potent against *En. cloacae* and *E. coli.* The range of MIC/MBC for Gram-positive bacteria was 26.3–344.8 µM and MBC at 52.6–689.6 µM, while for Gram-negative it was 26.3–378.5 µM, and MBC 52.6–757.0 µM, respectively showing any preference. In general, bacteria showed some similar sensitivity to compounds tested. Thus, the following bacterial strains *S. aureus, M. flavus, L. monocytogenes, P. aeruginosa* and *En. cloacae* responded to tested compounds in a similar way. *E. coli* emerged as the most sensitive bacterium meanwhile *P. aeruginosa* demonstrated the highest resistance rates.

The structure–activity relationship analysis revealed that substitution of the parent compound (**1**) had a positive effect on the antibacterial activity except for two cases (**9**, **15**, i.e., 4-F and 4-Br, respectively). As was mentioned above, the most active compound was the p-nitro derivative, which according to docking prediction at *E. coli* Mur B active site, showed lower energy. It seems that introducing NO_2_ at C-3 of phenyl core is favorable for antibacterial efficiency. Shifting this substituent to C-2 of benzene ring yielded the derivative **6** with reduced potency. Furthermore, beneficial for activity appeared to be the introduction of OH group in C2 of phenyl skeleton (**2**)**.** Displacement of the 2-OH group by 4-OH (**3**) had the same effect as the displacement of the 3-NO_2_ group. On the contrary, the replacement of 4-OH by 4-OMe group (**4**) had a positive effect. However, the presence of two methoxy groups at the 2,5-positions of the benzene ring (**5**) appears to be not positive with respect to the single methoxy substitution, most likely due to steric hindrances.

Concerning halide derivatives, it was observed that, as in the case of nitro derivatives, the displacement of the fluoro substituent from position 3 of the aromatic ring at position 4 led to a relevant reduction in efficiency. In addition, in the case of bromo-derivatives, position 3 (**14**) proved to be preferred for action with respect to 4-Br substitution (**15**).

Substitution at C-3 of the benzene ring appears to favor antibacterial activity. Regarding dichloro derivatives, the most active one seems to be the 2,6-dichloro derivative. Displacement of chlorine from position 6 to position 3 resulted in a small reduction in antibacterial activity while shifting of chlorine from position 6 to position 4 resulted in a greater decrease in antibacterial activity. In conclusion, the structure-action relationship study showed that antibacterial activity depends upon the nature of the substituent as well as on its position in the aromatic ring.

The compounds have also been studied for their antibacterial efficacy against resistant microbial strains (Table 2).

All compounds exhibited antibacterial activity against the tested resistant bacterial strains (MRSA, *Escherichia coli* and *Pseudomonas aeruginosa*), but to varying degrees. MIC of the derivatives was between 29.8–433.5 μM and MBC range between 59.6–867.0 μM. The antibacterial activity of these compounds can be presented in the following order: **12** > **15** > **10** > **4** > **8** > **5** > **3** > **1** > **14** > **13** > **2** > **11** > **7** > **9** > **6** > **STM** > **AMP**. Compared with ampicillin and streptomycin, compound **12** demonstrated the highest antibacterial action (MIC: 67.5–135.1 μM, MBC: 135.1–270.2 μM). The lowest efficiency was detected for compound **6.** The MIC range of streptomycin for the latter two resistant species was 86–171.9 μM and MBC 171.90–343 μM, while ampicillin exhibited only bacteriostatic activity (MIC) at 572.0 μM. Regarding MRSA, streptomycin exhibited only bacteriostatic activity, while ampicillin was not active at all.

All derivatives displayed better potency than ampicillin and some even superior activity to that of streptomycin. In particular, compounds **12** and **15** exhibited better effects than streptomycin and ampicillin against all resistant strains of bacteria. Most compounds (**1**, **3**, **4**, **5**, **7**, **8**, **10**, **12**, **13**, **15**) appeared to be more potent than streptomycin and ampicillin against the resistant strain of *E. coli*. In addition, compounds **4**, **8, 12**, **14** and **15** showed better activity than reference drugs against MRSA. The most sensitive bacterium to these compounds was *E. coli,* whereas methicillin-resistant *S. aureus* was found to be the most resistant.

In the case of resistant bacteria, it appears that in addition to the position of the substituents, the electron properties of the substituents play a significant role. In particular, it was observed that the first three best compounds contain electron acceptor groups.

The activity order of the tested compounds was different in resistant bacteria with respect to nonresistant. Compound **12,** the most potent against resistant strains, was one of the less active against nonresistant and vice versa. This is likely due to the different mechanisms of action in resistant bacteria, as they develop better defense mechanisms, producing chemicals and forming dense colonies, making them more difficult to be inhibited.

Five compounds (**2**, **7**, **8**, **13**, **14**) were also tested for inhibition of *P. aeruginosa* biofilm formation.

All examined compounds were able to reduce the biofilm-forming abilities of *P. aeruginosa* (Table 3). Percentage of reduction recorded after application of concentration equal to their MIC was above 50% for **8**, **13** and **14**, indicating good biofilm inhibiting potential. Compound **2** was the least active one in this assay, suggesting that inhibition of planktonic growth rather than biofilms is its mechanism of activity.

#### 2.3.2. Antifungal Activity

The compounds were then studied for their antimycotic activity, and results are displayed in Table 4.

All compounds showed very good antifungal activity. Particularly, the ranges of MIC and MFC were 27.7–578 μM and 55.4–1156.0 μM, respectively. The activity sequence can be presented as follows: **10** > **5** > **13 > 14 > 3** > **4** > **12** > **6** > **1** > **7** > **9** > **2** > **11** > **15** > **8** > **bifonazole** > **ketoconazole.** The most active compound was **10** with MIC 59.6–119.2 μM and MFC 119.2–238.4 μM, while the minimum effect was recorded for derivative **8** (MIC = 156.7–501.5 μM and MFC = 313.4–1003.0 μM). Bifonazole and ketoconazole, used as reference compounds, showed MIC in the range from 480 to 640 μM and from 380 to 4750 μM, respectively, whereas MFC were in the range from 640 to 800 μM and from 950 to 5700 μM, respectively. It is worth noting that all compounds exhibited much better action in comparison with references. In addition, all compounds indicated superior potency than reference drugs against *T. viride,* which was considered as the most sensitive fungus. Moreover, compounds **3**, **4**, **5**, **10**, **13**, **14** exhibited better potency compared to standard drugs against all fungal filaments. Compounds **1**, **2**, **6**, **7**, **9**, **12** displayed inhibitory actions on the growth of *A. fumigatus, A. niger, P. funiculosum* and *P. verrucosum. P. ochrochloron* emerged as the most resistant fungal species. Nevertheless, most derivatives were more efficacious than standard drugs against these fungi.

As in the antibacterial potency case, studies of structure–activity relationship revealed the dependence of the antifungal efficiency on the nature of substituents as well as on their position on the benzene skeleton. Thus, derivatives with 2,6-dichloro, 2,5-dimethoxy, 2,4-dichloro, 3-bromo, 4-hydroxy, 4-chloro, 4-methoxy, 3-nitro and 2-nitro substituents exhibited better antifungal activity than the parent compound, whereas the fluoro, 4-bromo, 2,3-dichloro and 2-hydroxy derivatives were less active than the parent compound (**1**). It seems that 4-chloro substitution is beneficial for antifungal activity (**10**), while its replacement with 4-fluoro (**9**) resulted in a significant reduction in activity, which decreased more upon replacement with 4-bromo (**15**). Among the dichloro-derivatives, 2,6-dichloro had the strongest effect. Displacement of 6-Cl at position 4 resulted in a compound with lesser action (**12**), while the shift of 6-Cl to position 3 (**11**) led to a larger reduction in activity. Considerable antifungal activity was also demonstrated by the 2,5-OMe derivative (**5**). The mono substitution with the methoxy group at C-4 induced a reduction in activity. With regard to nitro derivatives, the activity of the 3-NO_2_ compound was better compared to 2-NO_2_; the same was observed for antibacterial activity. From all the above-mentioned, it can be concluded that the antifungal-promoting substituents are 4-chloro, 2,5-dimethoxy, 2,6-dichloro and 3-bromo.

An important parameter for predicting membrane passage capability is the polar surface area (PSA) of a molecule, which is defined as the sum of the surface of polar atoms in a molecule. In particular, drugs with relatively small PSA < 140 A^2^ are almost completely absorbed through the small intestine with passive diffusion.

As can be seen from the above table (Table 5), no association was observed in any violation of the Lipinski rule. Therefore, all the compounds synthesized can very likely permeate the biological membranes as they have the appropriate physicochemical characteristics. The topological polar surface area (TPSA) of most compounds is less than 60 A^2^, which means that by oral administration, compounds are likely to be almost completely absorbed through the small intestine by passive diffusion.

### 2.4. Docking Studies

#### 2.4.1. Docking in *E. coli*-MurB

It was previously demonstrated that thiazolidinone derivatives act as MurB inhibitors [48,49,50,51]. MurB is an enzyme belonging to the superfamily of flavoproteins and plays a key role in cell wall biosynthesis as it participates in the second stage of synthesis of peptidoglycan, which is a crucial component of the bacterial cell wall. In particular, it catalyzes the final UDP-*N*-acetylmuramic acid (UDPMurNAc) formation step by reducing the NADPH-dependent enol pyruvate. Taking this into account and looking to study the mode of action of our compounds, a theoretical study of their binding to the *E. coli*-MurB enzyme active site (PDB: 2Q85) was accomplished. Results are presented in Table S2.

#### 2.4.2. Docking in Antifungal Targets

Prepared derivatives **1**–**15** and standard drug were subjected to docking with lanosterol 14α-demethylase from pathogenic yeast *C*. *albicans* (CYP51_Ca_) and dihydrofolate reductase to predict the probable mechanism of action using PDB: 5V5Z and PDB: 4HOF), respectively. The docking scores disclosed that CYP51_Ca_ was the most adequate to antifungal potency (Table S3).

### 2.5. Cellular Toxicity

**Figure 1:**
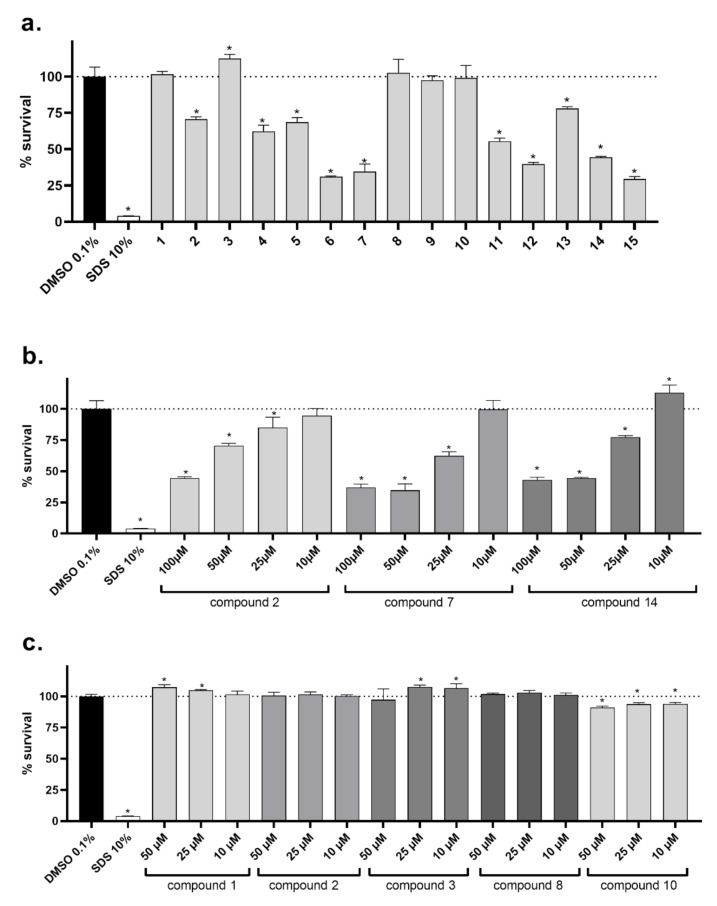
Cell toxicity of tested compounds on breast cancer cell lines MCF7/S0.5 and noncancerous cell line HK-2. (**a**) Screening at a concentration of 50 µM. (**b**) Dose-dependent toxicity in the case of the three active compounds, which were toxic at 50 µM. (**c**) Dose-dependent toxicity of five selected compounds that were not toxic at concentration 50 µM in HK-2 cell line. The statistical assay used was one-way ANOVA, * *p* < 0.001 vs. control (DMSO 0.1%). Results are presented as the mean of three independent experiments performed in triplicates.

In the last step, the potential cytotoxicity of all compounds was experimentally assessed. Based on our reported MICs and MBCs, an initial concentration of 50 µM was selected (Figure 1A). Five compounds (**6**, **7**, **12**, **14** and **15**) showed high toxicity in breast cancer cell line MCF7/S0.5, suggesting that their effect toward tested bacterial and fungal species can also be partly mediated by direct cytotoxicity. Importantly, the active derivatives **1**, **3**, **8** and **10**, which were active toward some bacterial species with MIC ranging from 26 to 63 µM, were not toxic to human cells neither at a concentration of 50 µM, which is a relatively high concentration. In the next experiments, we also tested the concentration-dependent toxicity of the three active compounds (**2**, **7** and **14**), which caused toxicity with different intensities at a concentration of 50 µM. As observed in Figure 1B, at lower concentrations, they were clearly non-toxic.

The toxicity of nitro-compounds (**6** and **7**) is a general but not always presented phenomenon [52,53,54] Nitro-group could be reduced likely in cells of different origin, and the process is associated with a highly reactive radical formation and tissue damage. These radicals are responsible not only for their cytotoxic effect but also for the antimicrobial properties of these compounds. Moreover, lipophilicity can contribute to better intracellular permeability and higher toxicity.

According to the literature, the majority of halogenated drugs are fluorine drugs, followed by chlorine ones, while bromine derivatives are rare [55]. Interestingly, our data on mono fluorine, chlorine and bromide compounds seems to fit in the general scheme, where fluorine and chlorine compounds are inert and hence non-toxic, while bromine compounds more easily release bromine and, in comparison, to previous ones are more toxic [56]. The toxicity shown by dichlorine-containing derivatives is peculiar and not understandable by the existed data. A possibility exists that may induce membrane permeability differently, thus accordingly altering their intracellular concentration. Another explanation may be focused on differences inherent to their molecular binding capacity within the cancer cells. At the same time, they can also target *P-*glycoprotein with various binding capacities, a fact that may cause limitation in a different degree to their achieved intracellular concentrations. However, this is only a hypothesis that deserves further investigation.

Even if noncancerous cells are in general more resistant toward cytotoxicity than cancer cells, the 5 most active compounds (**1**, **2**, **4**, **8** and **10**) were tested toward a renal cell line HK-2 for confirmation of safety (Figure 1C). As in the case of tumor cells, compounds **1**, **8** and **10** were non-toxic. In contrast to cancer cells, compound **2** did not decrease the proliferation of tested noncancerous cell lines. Contrarily, compound **10** was mildly cytotoxic.

## 3. Materials and Methods

### 3.1. Chemistry

#### 3.1.1. Synthesis of 5-Methylthiazol-2-yl chloroacetamide

To a solution of 2-amino-5-methylthiazole (0.3 g, 0.02 mol) and sodium carbonate (0.075 g, 0.008 mol) in 4.5 mL of anhydrous dimethylformamide (DMF) was added dropwise to a solution of chloroacetyl chloride (0.49 g, 0.033 mol) in 1.9 mL of DMF. The mixture was subjected to stirring for 3 h at 25 °C, then stopped, and the mixture was diluted with ice–water. Suction filtration yielded a solid, which was washed with H_2_O. It was recrystallized from C_2_H_5_OH. Yield: 85%, mp. 185−186 °C; I.R. (cm^−1^): 1709 (carbonyl), 3450 (N-H amide), 722 (Cl-C); ^1^H-NMR (DMSO-*d_6_* ; 300 MHz) δ ppm: 2.31 (s, 3H, methyl), 4.34 (s, 2H, CH_2_), 7.12 (s,1H, CH), 12.28 (s,1H, N-H amide).

#### 3.1.2. Synthesis of 2-(5-Methylthiazol-2-yl) thiazolidin-4-one

A solution of 5-methylthiazole 2-chloroacetamide (0.4 g, 0.05 mol) and ammonium thiocyanate (0.32 g, 0.01 mol) in C_2_H_5_OH 96% (20 mL) was refluxed for 1 h and then left overnight at room temperature. The obtained precipitate was recovered by filtration, washed with H_2_O d. It was recrystallized from C_2_H_5_OH. Yield: 76%, mp. 194–195 °C; I.R. (cm^−1^): 3160 (Ν-H amide), 1709 (carbonyl), 1622 (aromat.); ^1^H-NMR (DMSO-*d_6_* ; 300 MHz) δ ppm: 2.01 (s, 3H, CH_3_), 4.08 (s, 2H, CH_2_), 7.31 (s, 1H, CH), 12.21 (s, 1H, N-H amide).

#### 3.1.3. General Procedure for the Synthesis of 5-Benzyliden-2-(5-methylthiazol-2-ylimino) thiazolidin-4-one

To a solution of 2-[5-methylthiazol-2-yl)-thiazolidin-4-one (0.35 g, 0.001 mol) in CH_3_COOH (17 mL) buffered with CH_3_COONa (0.16 g, 0.002 mol,) was added an adequate benzaldehyde (0.0015 mol). The mixture was refluxed for 4 h and then drained on ice-H_2_O. The obtained solid was recovered by filtration and washed with H_2_O. It was recrystallized from absolute C_2_H_5_OH or dioxane.

##### 5-benzyliden -2-(5-methylthaizol-2-ylimino) thiazolidin-4-one (1)

This compound was published in our previous paper [48].

##### 2-(5-methylthiazol-2-ylimino)-5-(2-hydroxybenzyliden) thiazolidin-4-one (2)

Yield: 98%, mp. 215–216 °C (dioxane); IR (cm^−1^): 1720 (carbonyl), 2352 (-N-H amide), 2720 (OH); ^1^H-NMR (DMSO-*d_6_* ; 300 MHz) δ: 2.33 (s, 3H, -CH_3_), 6.74 (s, 1H, OH), 7.13–7.26 (m, 3H, Ar), 7.38 (s, 1H, -CH-N), 7.42–7.51 (m, 2H, Ar, =C-H) 7.88 (s, 1H, -N-H); ^13^C-NMR **(**CDCl_3_; 125 MHz) δ ppm: δ: 11.81, 116.02, 116.75, 117.73, 121.22, 128.95, 192.21, 130.84, 137.01, 143.02, 157.13, 158.24, 171.02, 174.23. Anal. Calcd. for C_14_H_11_N_3_O_2_S_2_ (%): C, 52.98; H, 3.49; N, 13.24; O, 10.08; S, 20.21. Found (%): C, 52.95; H, 3.46; N, 13.22.

##### 2-(5-methylthiazol-2-ylimino)-5-(4-hydroxybenzyliden) thiazolidin-4-one (3)

Yield: 42.8%, mp. 210–211 °C (dioxane); IR. (cm^−1^): 1738 (carbonyl), 2360 (-N-H amide), 2725 (OH); ^1^H-NMR (DMSO-*d_6_* ; 300 MHz) δ. 2.34 (s, 3H, -CH_3_), 6.93 (s, 1H, Ar), 7.21–7.69 (m, 5H, 3Ar, -CH thiazole, =C-H), 10.31 (s, 1H, -O-H), 11.96 (s, 1H, -N-H); ^13^C-NMR **(**CDCl_3_; 125 MHz) δ ppm: 11.82, 115.03, 116.12, 127.91, 130.68, 131.12, 138.71, 143.03, 157.77, 158.02, 171.03, 174.19. Anal. Calcd. for C_14_H_11_N_3_O_2_S_2_ (%): C, 52.98; H, 3.49; N, 13.24; O, 10.08; S, 20.21. Found (%): C, 52.96; H, 3.44; N, 13.23.

##### 2-(5-methylthiazol-2-ylimino)-5-(4-methoxybenzyliden) thiazolidin-4-one (4)

Yield: 35%, mp. 205–206 °C (dioxane); IR (cm^−1^): 1720 (carbonyl), 2380 (-N-H amide); ^1^H-NMR (DMSO-*d_6_* ; 300 MHz) δ: 2.33 (s, 3H, -CH_3_), 3.88 (s, 3H, -CH_3_O), 6.97–7.23 (m, 3H, -CH thiazole, 2 Ar), 7.34–7.66 (m,3H, =C-H, Ar), 7.79 (s,1H, NH); ^13^C-NMR **(**CDCl_3_; 125 MHz) δ ppm: 11.80, 58.86, 114.23, 116.11, 127.91, 130.05, 131.12, 138.64, 143.15, 158.02, 159.83, 171.03, 174.22. Anal. Calcd. For C_15_H_13_N_3_O_2_S_2_ (%): C, 54.36; H, 3.95; N, 12.68; O, 9.66; S, 19.35. Found (%): C, 54.35; H, 3.96; N, 12.61

##### 2-(5-methylthiazol-2-ylimino)-5-(2,5-dimethoxybenzyliden) thiazolidin-4-one (5)

Yield: 30%, mp. 200–201 °C (dioxane), R_f_ = 0.55 (PhCH_3:_C_2_H_5_OH 8:2), IR (cm^−1^): 1715 (carbonyl), 2406 (-N-H amide); ^1^H-NMR (DMSO-*d_6_* ; 300 MHz) δ: 2.34 (s, 3H, -CH_3_), 3.75 (s, 6H, -CH_3_-O), 6.98–7.22 (m, 3H, 2 Ar, -CH thiazole), 7.32–7.45 (m, 2H, =C-H, Ar), 7.83 (s, 1H, -N-H). ^13^C-NMR **(**CDCl_3_; 125 MHz) δ ppm: 19.24, 56.2, 57.12, 11.17, 114.36, 115.93, 117.96, 118.85, 143.18, 150.23, 151.11, 151.96, 152.87, 158.34, 168.05, 173.67. Anal. Calcd. For C_16_H_15_N_3_O3S_2_ (%): C, 53.17; H, 4.18; N, 11.63; O, 13.28; S, 17.74. Found (%): C, 53.15; H, 4.14; N, 11.61.

##### 2-(5-methylthiazol-2-ylimino)-5-(2-nitrobenzyliden) thiazolidin-4-one (6)

This compound was published in our previous paper [48].

##### 2-(5-methylthiazol-2-ylimino)-5-(3-nitrobenzyliden) thiazolidin-4-one (7)

This compound was published in our previous paper [48].

##### 2-(5-methylthiazol-2-ylimino)-5-(3-fluorobenzyliden) thiazolidin-4-one (8)

This compound was published in our previous paper [48].

##### 2-(5-methylthiazol-2-ylimino)-5-(4-fluorobenzyliden) thiazolidin-4-one (9)

Yield: 39%, mp. 178–179 °C (dioxane), R_f_ = 0.77 (PhCH_3:_C_2_H_5_OH 8:2), IR (cm^−1^): 1597 (carbonyl), 3050 (-N-H amide); ^1^H-NMR (DMSO-*d_6_*; 300 MHz) δ: 2.36 (s, 3H, -CH_3_), 7.31–7.45 (μ, 3H, H-3, H-5, -CH thiazole), 7.63–7.75 (m, 3H, 2 Ar, =C-H), 12.55 (s, 1H, -N-H). ^13^C-NMR **(**CDCl_3_; 125 MHz) δ ppm: 11.81, 115.23, 116.71, 127.51, 130.04, 132.12, 138.64, 143.15, 158.72, 162.04, 171.03, 174.21. Anal. Calcd. For C_14_H_10_FN_3_OS_2_ (%): C, 52.65; H, 3.16; F, 5.95; N, 13.16; O, 5.01; S, 20.08. Found (%): C, 52.62; H, 3.15; N, 13.12.

##### 2-(5-methylthiazol-2-ylimino)-5-(4-chlorobenzyliden) thiazolidin-4-one (10)

This compound was published in our previous paper [48].

##### 2-(5-methylthiazol-2-ylimino)-5-(2,3-dichlorobenzyliden) thiazolidin-4-one (11)

Yield: 61.7%, mp. 249–250 °C (dioxane), R_f_ = 0.60 (PhCH_3:_C_2_H_5_OH 8:2), IR: (cm^−1^, Nujol), 1709 (carbonyl), 2724 (N-H amide), 721 (-C-Cl); ^1^H-NMR (DMSO-*d_6_* ; 300 MHz) δ: 2.36 (s, 3H, -CH_3_), 7.33 (s, 1H, -CH thiazole), 7.56–7.61 (m, 3H, Ar), 7.71 (s, 1H, =C-H), 7.79 (s, 1H, -N-H). ^13^C-NMR **(**CDCl_3_; 125 MHz) δ ppm: 19.31, 114.17, 125.12, 128.14, 128.92, 129.76, 133.16, 141.11, 143.91, 150.44, 150.48, 159.67, 168.11, 173.26. Anal. Calcd. For C_14_H_9_Cl_2_N_3_OS_2_ (%): C, 45.41; H, 2.45; Cl, 19.15; N, 11.35; O, 4.32; S, 17.32. Found (%): C, 45.38; H, 2.42; N, 11.31.

##### 2-(5-methylthiazol-2-ylimino)-5-(2,4-dichlorobenzyliden) thiazolidin-4-one (12)

Yield: 57.7%, mp. 260–261 °C (dioxane), R_f_ = 0.77 (PhCH_3:_C_2_H_5_OH 8:2), IR: (cm^−1^, Nujol), 1729 (carbonyl), 3049 (N-H amide), 720 (-Cl-C); ^1^H-NMR (DMSO-*d_6_* ; 300 MHz) δ: 2.36 (s, 3H, -CH_3_), 7.43 (s, 1H, -CH thiazole), 7.53–7.79 (m, 4H, 3 Ar, =C-H), 12.73 (s, 1H, -N-H). ^13^C-NMR **(**CDCl_3_; 125 MHz) δ ppm: 11.94, 116.15, 125.27, 126.03, 129.11, 130.14, 131.17 (2C), 136.27, 138.56, 143.33, 158.26, 168.55, 174.28. Anal. Calcd. For C_14_H_9_Cl_2_N_3_OS_2_ (%) : C, 45.41; H, 2.45; Cl, 19.15; N, 11.35; O, 4.32; S, 17.32. Found (%): C, 45.36; H, 2.43; N, 11.30.

##### 2-(5-methylthiazol-2-ylimino)-5-(2,6-dichlorobenzyliden) thiazolidin-4-one (13)

Yield: 94.6%, mp. 225–226 °C (dioxane), R_f_ = 0.60 (PhCH_3:_C_2_H_5_OH 8:2), IR: (cm^−1^, Nujol), 1600 (carbonyl), 2359 (-N-H amide), 722 (-Cl-C); ^1^H-NMR (DMSO-*d_6_* ; 300 MHz) δ: 2.36 (s, 3H, -CH_3_), 7.33 (s, 1H, -CH thiazole), 7.54–7.60 (m, 3H, 3 Ar), 7.71 (s, 1H, =C-H), 7.77 (s, 1H, -N-H). ^13^C-NMR **(**CDCl_3_; 125 MHz) δ ppm: 11.17, 116.14, 126.20, 127.85 (2C), 129.14 (2C), 130.52, 132.78, 135.22, 138.01, 143.29, 157.93, 168.73, 174.26. Anal. Calcd. For C_14_H_9_Cl_2_N_3_OS_2_ (%): C, 45.41; H, 2.45; Cl, 19.15; N, 11.35; O, 4.32; S, 17.32. Found (%): C, 45.37; H, 2.40; N, 11.31.

##### 2-(5-methylthiazol-2-ylimino)-5-(3-bromobenzyliden) thiazolidin-4-one (14)

Yield: 28.8%, mp. 220–221 °C (dioxane), R_f_ = 0.57 (PhCH_3:_C_2_H_5_OH 8:2), IR: (cm^−1^, Nujol), 1704 (carbonyl), 2356 (N-H amide); ^1^H-NMR (DMSO-*d_6_* ; 300 MHz) δ: 2.36 (s, 3H, -CH_3_), 7.43 (s, 1H, -CH thiazole), 7.48–7.83 (m, 4H, Ar), 7.89 (s, 1H, =C-H), 12.53 (s, br, 1H, -N-H). ^13^C-NMR **(**CDCl_3_; 125 MHz) δ ppm: 11.15, 116.11, 123.28, 126.87, 128.53, 129.15, 130.77, 131.93, 137.18, 138.06, 142.61, 158.14, 168.79, 174.12. Anal. Calcd. For C_14_H_10_BrN_3_OS_2_ (%): C, 44.22; H, 2.65; Br, 21.01; N, 11.05; O, 4.21; S, 16.86. Found (%): C, 44.18; H, 2.61; N, 10.97.

##### 2-(5-methylthiazol-2-ylimino)-5-(4-bromobenzyliden) thiazolidin-4-one (15)

Yield: 57.0%, mp. 259–260 °C (dioxane), R_f_ = 0.53 (PhCH_3:_C_2_H_5_OH 8:2), IR: (cm^−1^, Nujol), 1711 (carbonyl), 3249 (-N-H amide). ^1^H-NMR**:** (δ, DMSO-d_6,_ 300 MHz 2.2 (s, 3H, -CH_3_), 7.36 (d, 2H, -CH thiazole, =CH), 7.55–7.77 (m, 4-H, Ar), 12.67 (s, 1H, -N-H). ^13^C-NMR **(**CDCl_3_; 125 MHz) δ ppm: 11.16, 116.10, 123.42, 128.05 (2C), 130.19 (2C), 131.93, 135.18, 138.72, 143.01, 158.13, 168.82, 174.04. Anal. Calcd. For C_14_H_10_BrN_3_OS_2_ (%): C, 44.22; H, 2.65; Br, 21.01; N, 11.05; O, 4.21; S, 16.86. Found (%): C, 44.20; H, 2.63; N, 10.98.

### 3.2. Biological Evaluation

#### 3.2.1. Antibacterial Action

Bacterial strains utilized include Gram-negative: *Salmonella typhimurium*, (ATCC 13311) *Pseudomonas aeruginosa* (ATCC 27853), *Escherichia coli* (ATCC 35210), *Enterobacter cloacae* (ATCC 35030) and Gram-positive bacteria: *Micrococcus flavus* (ATCC 10240), *Bacillus cereus* (isolated clinically), *Staphylococcus aureus* (ATCC 6538) and *Listeria monocytogenes* (NCTC 7973) bacteria. Pathogens were provided from the Mycological Laboratory, Institute for Biological Research “Siniša Stankovic” Belgrade. Resistant strains used were MRSA, *E. coli* and *P. aeruginosa.*

The MIC/MBC were effectuated utilizing microdilution assay as previously described [57,58].

#### 3.2.2. Methicillin-resistant *Staphylococcus aureus* (MRSA)

This strain is isolated from cows with subclinical mastitis. Milk samples were streaked onto Columbia agar plates (Torlak, Beograd, Serbia) containing 5% sheep blood, Baird-Parker agar plates (HiMedia, Mumbai, Maharashtra, India) and chromogenic culture media (chromID MRSA, bioMérieux, Marcy l’Etoile, France). After incubation at 37 °C for 24 h, the colonies were presumptively identified according to morphological features, pigment production, Gram staining results, catalase and oxidase tests, type of hemolysis and characteristic growth on Baird-Parker agar plates (HiMedia, Mumbai, Maharashtra, India), BP agar and chromogenic culture media (chromID MRSA, bioMérieux, Durham, NC, USA) chromID MRSA. Suspected colonies of *S. aureus* on the blood agar and green colonies on chromogenic media were transferred to individual plates to obtain pure cultures. The identification was confirmed using a BBL Crystal G/P ID kit (Becton Dickinson, Nairobi, Kenya). Antimicrobial susceptibility testing was performed by the disk diffusion method with 30 μg cefoxitin discs (Rosco, Taastrup, Denmark) in accordance with the Clinical and Laboratory Standard Institute recommendations. All isolated strains of *S. aureus* were tested for the presence of penicillin-binding protein (PBP2) with latex agglutination tests (Slidex MRSA detection, bioMérieux, Zhujiang, New Town, China ). *Staphylococcus aureus* ATCC 25923 was used as the control strain. All isolates were tested for the presence of the *mecA* gene by PCR [59].

#### 3.2.3. *E. coli*

Samples of rectal swabs, feces and intestines from diseased pigs were taken. In order to isolate *E. coli* strains, the following nutrition media were used: MacConkey agar (Torlak, Beograd, Serbia), Columbia agar (Torlak, Beograd, Serbia) with 5% defibrinated sheep blood and brilliant green agar (Torlak). For the identification of the isolated strains, laboratory tests with the following nutritious media and reagents were performed: Simmons citrate agar (Torlak), MR/VP broth (Torlak), Christensen urea agar (Torlak, Beograd, Serbia), peptone water for the indole test (Torlak), catalase and oxidase, triple sugar agar (Torlak), as well as identification systems BBL crystal entero/nonfermenter ID kit (Becton Dickinson Nairobi, Kenya,). Sensitivity studies on the isolated bacteria were completed by the disc diffusion method on Mueller–Hinton agar with the use of antibiogram discs (Bioanalyse, Ankara, Turkey) and tablets (Torlak) for the following antibiotics: penicillin, ampicillin, amoxicillin, tetracycline, neomycin, gentamicin, colistin, ceftriaxone, sulfamethoxazole with trimethoprim, enrofloxacin and florfenicol. All isolated *E. coli* strains were resistant to all tested antibiotics with the exception of enrofloxacin, colistin and florfenicol [60].

#### 3.2.4. *Pseudomonas aeruginosa*

The strains were isolated from cats and dogs. Samples were inoculated on Columbia agar plates (Torlak, Serbia) containing 5% sheep blood, nutrition agar (HiMedia) and MacConkey agar (Torlak) and incubated under aerobic conditions at temperatures of 37 °C and 42 °C for 24 h. Pure cultures were identified on the basis of morphological and biochemical characteristics. For identification of pigment production, subcultivation on the corresponding medium was carried out. Identification was confirmed using a BBL crystal entero/nonfermenter ID kit (Becton Dickinson). Sensitivity studies were completed by the disc diffusion method on Mueller–Hinton agar with the use of antibiogram discs (Bioanalyse) and tablets (Torlak) for the following antibiotics: penicillin G, ampicillin, amoxicillin, tetracycline, neomycin, gentamicin, ceftriaxone, sulfamethoxazole with trimethoprim, enrofloxacin and florfenicol. All isolated *Pseudomonas aeruginosa* strains were resistant to all tested antibiotics with the exception of enrofloxacin and florfenicol [60].

#### 3.2.5. Inhibition of Biofilm Formation

The method was performed as described by us [61] with some modifications. Briefly, *P. aeruginosa* resistant strain was incubated with MIC and subMIC of tested compounds in tryptic soy broth enriched with 2% glucose at 37 °C for 24 h. After 24 h, each well was washed twice with sterile PBS (phosphate-buffered saline, pH 7.4) and fixed with methanol for 10 min. Methanol was then removed, and the plate was air-dried. Biofilm was stained with 0.1% crystal violet (Bio-Merieux, Marcy l’Etoile, France) for 30 min. Wells were washed with water, air dried, and 100 μL of 96% ethanol (Zorka, Sabac, Serbia) was added. The absorbance was read at 620 nm on a Multiskan™ FC microplate photometer, Thermo Scientific™. The percentage of inhibition of biofilm formation was calculated by the formula:

[(A_620_ control − A_620_ sample)/A_620_ control) × 100]

#### 3.2.6. Antifungal Activity

For the antifungal bioassays, eight fungi were used: *Aspergillus niger* (ATCC 6275), *Aspergillus ochraceus* (ATCC 12066), *Aspergillus fumigatus* (human isolate), *Aspergillus versicolor* (ATCC 11730), *Penicillium funiculosum* (ATCC 36839), *Penicillium ochrochloron* (ATCC 9112), *Trichoderma viride* (IAM 5061), *Penicillium verrucosum var. cyclopium* (food isolate). The organisms were obtained from the Mycological Laboratory, Department of Plant Physiology, Institute for Biological Research “Siniša Stankovic”, Belgrade, Serbia. All experiments were performed in duplicate and repeated three times [62,63].

### 3.3. Statistical Analysis

All tests were performed three times, and the values were determined as standard deviation (SD) and mean values. A one-way ANOVA test was allowed to determine variance analysis with Tukey HSD Test (0.05 levels). The analysis was executed with the help of SPSS statistics software (version 18).

### 3.4. Cytotoxicity

CellTiter 96^®^ aqueous nonradioactive cell proliferation assay (Promega, Madison, WI, USA) was performed to evaluate the in vitro effects of evaluated compounds in breast adenocarcinoma MCF7/S0.5 (parental MCF7 cells adapted to low-sera conditions) and human kidney immortalized cell line HK-2. The employed method uses the bioreduction of tetrazolium salt of MTS (3-(4,5-dimethylthiazol-2-yl)-5-(3-carboxymethoxyphenyl)-2-(4-sulfophenyl)-2H-tetrazolium) into a colored formazan with an absorbance peak maximum at wavelength 490 nm. Only viable cells are able to metabolize the compound. Experiments were conducted in accordance with manufacturer guidelines. Briefly, cells were treated with the test compounds, negative control (SDS 10%) or vehicle (DMSO 0.1%) for 48 h in 96-wells plates. At the end of the treatment, 20 μL of MTS reagent was added to each well and incubated for a further 3 h prior to absorbance measurement using a plate reader (Hidex Sense Beta Plus plate reader, Hidex, Turku, Finland). MCF7/S0.5 cell line was cultivated in DMEM/F-12 media w/o phenol red supplemented with 1% FBS and insulin 6 ng/mL. HK-2 cells were cultivated in DMEM with high-glucose and L-glutamine, supplemented with 10% FBS. Results are expressed as the relative cell viability, considering the vehicle to have 100% viability.

## 4. Conclusions

Fifteen newly designed and synthesized 5-benzyliden-2-(5-methylthiazol- 2-ylimino)thiazolidin-4-ones exhibited significant inhibition of the growth of a wide spectrum of Gram-positive, Gram-negative bacteria and fungi. The majority of derivatives were more efficient than the standard antibacterial drug ampicillin. Compounds **2–8**, **10**, **11**, **13** and **14** also exhibited higher potency than streptomycin. The most sensitive bacterium was *E. coli*; meanwhile, *P. aeruginosa* demonstrated the highest resistance rates.

Furthermore, some of the compounds showed better or comparable potency compared to streptomycin, with the most potent among them being compound **8** (15 and 7-fold more active than ampicillin and streptomycin, respectively)**.** It was observed that, among the Gram-negative bacteria, the most sensitive to the tested compounds was *E. coli*, while *S. typhimurium* was the most resistant one. Regarding the Gram-positive bacteria, the most sensitive one was *B. cereus,* while *L. monocytogenes* were found to be the most resistant bacterium.

Furthermore, all compounds exhibited antibacterial potency against the tested resistant bacterial strains displaying better efficacy than ampicillin and some of them (**12** and **15**) even higher than streptomycin. Compounds **1**, **3**, **4**, **5**, **7**, **8**, **10**, **12**, **13**, **15** appeared to be more potent than streptomycin and ampicillin against the resistant strain of *E. coli,* which was the most sensitive, while some of them (**4**, **8**, **12**, **14** and **15)** exhibited better activity than reference drugs against methicillin-resistant *Staphylococcus aureus*, the most resistant strain. Compounds **8**, **13** and **14** exhibited significant antibiofilm activity.

As regards antifungal activity, most of the examined compounds displayed better potency than reference drugs, ketoconazole and bifonazole. Thus, the most active compound **10** was found to be 4–8 and 7–13-fold more active than bifonazole and ketoconazole, respectively.

Docking analysis to *E. coli* MurB indicated a probable involvement of MurB inhibition in the antibacterial mechanism of compounds tested, while docking to 14α-lanosterol demethylase (CYP51) and tetrahydrofolate reductase of *Candida albicans* indicated a probable implication of CYP51 reductase at the antifungal activity of the compounds.

Finally, toxicity prediction revealed that compounds are not toxic. In addition, according to the prediction of physicochemical parameters for the passage through biological membranes, they will likely be absorbed via passive diffusion when given orally. Anyway, their toxicity toward a human cell line was experimentally assessed at a relatively high concentration of 50 µM. Several active compounds were not toxic even at this high concentration.

## Figures and Tables

**Table 1:** Antibacterial action of the thiazolidinone derivatives (MIC/MBC are in μM).

Compounds	R	MIC/MBC	*B.c.*	*S.a.*	*M.f.*	*L.m.*	*P.a.*	*En.cl.*	*E.c.*	*S.t.*
**1**	**H**	**MIC**	33.2 ± 0.03	265.8 ± 0.03	265.8 ± 0.04	265.8 ± 0.06	265.8 ± 0.08	265.8 ± 0.03	33.2 ± 0.01	265.8 ± 0.05
		**MBC**	66.4 ± 0.02	531.6 ± 0.4	531.6 ± 0.6	531.6 ± 0.3	531.6 ± 0.5	531.6 ± 0.5	66.4 ± 0.03	531.6 ± 0.3
**2**	**2-OH**	**MIC**	94.6 ± 0.03	94.6 ± 0.05	94.6 ± 0.02	94.6 ± 0.01	94.6 ± 0.03	94.6 ± 0.03	94.6 ± 0.03	94.6 ± 0.02
		**MBC**	189.2 ± 005	189.2 ± 0.08	189.2 ± 0.01	189.2 ± 0.05	189.2 ± 0.02	189.2 ± 0.04	189.2 ± 0.04	189.2 ± 0.03
**3**	**4-OH**	**MIC**	63.0 ± 003	126.1 ± 0.06	189.2 ± 0.02	126.1 ± 0.04	378.5 ± 0.8	126.1 ± 0.05	63.0 ± 0.02	126.1 ± 0.02
		**MBC**	126.1 ± 0.03	252.3 ± 0.05	378.5 ± 0.6	252.3 ± 0.03	757.0 ± 0.8	252.3 ± 0.05	126.1 ± 0.03	252.3 ± 0.03
**4**	**4-OCH_3_**	**MIC**	30.2 ± 0.04	120.8 ± 0.05	120.8 ± 0.03	120.8 ± 0.05	261.6 ± 0.05	120.8 ± 0.06	30.2 ± 0.01	120.8 ± 0.05
		**MBC**	60.4 ± 006	241.6 ± 0.06	241.6 ± 0.02	241.6 ± 0.06	483.3 ± 0.6	241.6 ± 0.06	60.4 ± 0.04	241.6 ± 0.04
**5**	**2,5-di-OCH_3_**	**MIC**	96.9 ± 0.06	193.9 ± 0.05	193.9 ± 0.03	193.9 ± 0.04	193.9 ± 0.06	96.9 ± 0.04	193.9 ± 0.03	193.9 ± 0.03
		**MBC**	193.9 ± 0.05	387.8 ± 0.3	387.8 ± 0.4	387.8 ± 0.3	387.8 ± 0.4	193.9 ± 0.08	387.8 ± 0.4	387.8 ± 0.4
**6**	**2-NO_2_**	**MIC**	57.8 ± 0.03	115.6 ± 0.04	231.2 ± 0.08	115.6 ± 0.03	115.6 ± 0.05	115.6 ± 0.04	115.6 ± 0.04	231.2 ± 0.06
		**MBC**	115.6 ± 0.03	115.6 ± 0.03	462.4 ± 0.03	231.2 ± 0.02	231.2 ± 0.08	231.2 ± 0.04	231.2 ± 0.05	462.4 ± 0.4
**7**	**3-NO_2_**	**MIC**	86.7 ± 0.02	86.7 ± 0.02	86.7 ± 0.02	86.7 ± 0.02	86.7 ± 0.05	43.3 ± 0.03	43.3 ± 0.03	86.7 ± 0.05
		**MBC**	173.4 ± 0.01	173.4 ± 0.06	173.4 ± 0.04	173.4 ± 0.03	173.4 ± 0.03	86.7 ± 0.02	86.7 ± 0.02	173.4 ± 0.06
**8**	**3-F**	**MIC**	125.4 ± 0.03	125.4 ± 0.08	125.4 ± 0.04	125.4 ± 0.04	125.4 ± 0.04	62.7 ± 0.01	62.7 ± 0.01	125.4 ± 0.06
		**MBC**	250.8 ± 0.06	250.8 ± 0.06	250.8 ± 0.06	250.8 ± 0.06	250.7 ± 0.05	125.4 ± 0.03	125.4 ± 0.03	250.8 ± 0.08
**9**	**4-F**	**MIC**	344.8 ± 0.5	344.8 ± 0.3	344.8 ± 0.2	344.8 ± 0.3	344.8 ± 0.6	125.4 ± 0.05	125.4 ± 0.03	344.8 ± 0.4
		**MBC**	689.6 ± 0.8	689.6 ± 0.6	689.6 ± 0.3	689.6 ± 0.8	689.6 ± 0.8	250.7 ± 0.07	250.7 ± 0.03	689.6 ± 0.6
**10**	**4-Cl**	**MIC**	26.3 ± 0.01	89.4 ± 0.03	178.8 ± 0.04	89.4 ± 0.07	357.6 ± 0.4	178.8 ± 0.03	26.3 ± 0.02	357.6 ± 0.6
		**MBC**	52.6 ± 0.01	178.8 ± 0.02	357.6 ± 0.3	178.8 ± 0.08	715.3 ± 0.8	357.6 ± 0.4	52.6 ± 0.03	715.3 ± 0.8
**11**	**2,3-di-Cl**	**MIC**	189.2 ± 0.06	189.2 ± 0.06	189.2 ± 0.05	189.2 ± 0.06	189.2 ± 0.06	94.6 ± 0.02	94.6 ± 0.05	189.2 ± 0.05
		**MBC**	378.4 ± 0.5	378.4 ± 0.6	378.4 ± 0.06	378.4 ± 0.8	378.4 ± 0.5	189.2 ± 0.03	189.2 ± 0.04	378.4 ± 0.3
**12**	**2,4-di-Cl**	**MIC**	135.1 ± 0.03	135.1 ± 0.05	135.1 ± 0.04	270.2 ± 0.4	270.2 ± 0.3	270.2 ± 0.4	135.1 ± 0.02	270.2 ± 0.3
		**MBC**	270.2 ± 0.3	270.2 ± 0.4	270.2 ± 0.4	540.5 ± 0.5	540.5 ± 0.4	540.5 ± 0.6	270.2 ± 0.3	540.5 ± 0.8
**13**	**2,6-di-Cl**	**MIC**	40.5 ± 0,004	81.0 ± 0.06	81.0 ± 0.03	81.0 ± 0.05	162.1 ± 0.05	162.1 ± 0.05	81.0 ± 0.03	162.1 ± 0.05
		**MBC**	81.0 ± 0.06	324.2 ± 0.4	162.1 ± 0.02	162.1 ± 0.06	324.2 ± 0.5	324.2 ± 0.3	162.1 ± 0.04	324.2 ± 0.5
**14**	**3-Br**	**MIC**	78.9 ± 0.06	78.9 ± 0.05	78.9 ± 0.03	78.9 ± 0.06	157.9 ± 0.07	78.9 ± 0.03	78.9 ± 0.05	157.9 ± 0.05
		**MBC**	157.9 ± 0.05	157.9 ± 0.04	157.9 ± 0.05	157.9 ± 0.05	315.8 ± 0.5	157.9 ± 0.04	157.9 ± 0.06	315.8 ± 0.3
**15**	**4-Br**	**MIC**	78.9 ± 0.01	342.1 ± 0.5	342.1 ± 0.3	342.1 ± 0.5	342.1 ± 0.6	342.1 ± 0.6	78.9 ± 0.03	342.1 ± 0.4
		**MBC**	157.9 ± 0.05	684.2 ± 0.8	684.2 ± 0.8	684.2 ± 0.8	684.2 ± 0.8	684.2 ± 0.8	157.9 ± 0.04	684.2 ± 0.9
**STM**		**MIC**	43.0 ± 0.04	172 ± 0.3	86 ± 0.02	258 ± 0.4	172 ± 0.3	43 ± 0.04	172 ± 0.3	172 ± 0.3
		**MBC**	86 ± 0.05	344 ± 0.5	172 ± 0.3	516 ± 0.5	344 ± 0.4	86 ± 0.05	344 ± 0.4	344 ± 0.4
**AMP**		**MIC**	248 ± 0.3	248 ± 0.4	248 ± 0.3	372 ± 0.3	744 ± 0.6	248 ± 0.6	372 ± 0.4	248 ± 0.4
		**MBC**	372 ± 0.4	372 ± 0.5	372 ± 0.6	744 ± 0.6	1240 ± 0.8	372 ± 0.4	492 ± 0.6	492 ± 0.3

*S.t.—S. typhimurium, L.m.—L. monocytogenes, En.c.—En. cloacae, B.c.—B. cereus, M.f.—M. flavus, E.c.—E. coli, P.a.—P. aeruginosa, S.a.—S. aureus*, AMP—ampicillin, STM—streptomycin. MIC and MBC in μM.

**Table 2:** Antibacterial potency results of the thiazole derivatives (MIC and MBC in μM) towards resistant strains.

Νο.	MIC/MBC	*MRSA*	*P.a.*	*E.c.*
**1**	**MIC**	255.7 ± 0.04	122.8 ± 0.1	33.2 ± 0.03
	**MBC**	531.5 ± 0.08	265 ± 0.3	66.4 ± 0.02
**2**	**MIC**	173.4 ± 0.05	173.4 ± 0.05	173.4 ± 0.04
	**MBC**	346.8 ± 0.03	346.8 ± 0.4	346.8 ± 0.10
**3**	**MIC**	189.2 ± 0.2	189.2 ± 0.4	31.5 ± 0.05
	**MBC**	378.5 ± 0.03	378.5 ± 0.02	63.0 ± 0.04
**4**	**MIC**	120.8 ± 0.08	120.8 ± 0.3	60.4 ± 0.04
	**MBC**	241.6 ± 0.02	241.6 ± 0.03	120.8 ± 0.05
**5**	**MIC**	193.9 ± 0.06	96.9 ± 0.10	96.9 ± 0.02
	**MBC**	387.8 ± 0.03	193.9 ± 0.5	193.9 ± 0.04
**6**	**MIC**	433.5 ± 0.06	433.5 ± 0.03	433.5 ± 0.4
	**MBC**	867.0 ± 0.02	867.0 ± 0.3	867.0 ± 0.10
**7**	**MIC**	289.0 ± 0.03	144.5 ± 0.06	144.5 ± 0.02
	**MBC**	578.0 ± 0.04	289.0 ± 0.03	289.0 ± 0.4
**8**	**MIC**	125.4 ± 0.02	125.4 ± 0.08	125.4 ± 0.05
	**MBC**	250.8 ± 0.03	250.8 ± 0.01	250.8 ± 0.06
**9**	**MIC**	219.4 ± 0.08	219.4 ± 0.3	219.4 ± 0.04
	**MBC**	438.8 ± 0.03	438.8 ± 0.10	438.8 ± 0.02
**10**	**MIC**	178.8 ± 0.06	89.4 ± 0.03	29.8 ± 0.02
	**MBC**	357.6 ± 0.05	178.8 ± 0.02	59.6 ± 0.10
**11**	**MIC**	189.2 ± 0.06	189.2 ± 0.5	189.2 ± 0.03
	**MBC**	378.4 ± 0.04	378.4 ± 0.03	378.4 ± 0.02
**12**	**MIC**	135.1 ± 0.08	67.5 ± 0.09	67.5 ± 0.06
	**MBC**	270.2 ± 0.06	135.1 ± 0.3	135.1 ± 0.4
**13**	**MIC**	270.2 ± 0.05	135.1 ± 0.3	135.1 ± 0.02
	**MBC**	540.5 ± 0.06	270.2 ± 0.5	270.2 ± 0.03
**14**	**MIC**	131.6 ± 0.04	131.6 ± 0.06	263.2 ± 0.06
	**MBC**	263.2 ± 0.02	263.2 ± 0.04	526.4 ± 0.02
**15**	**MIC**	157.9 ± 0.03	78.9 ± 0.02	78.9 ± 0.01
	**MBC**	215.8 ± 0.04	157.9 ± 0.10	157.9 ± 0.05
**Streptomycin**	**MIC**	171.9 ± 0.02	86 ± 0.3	171.9 ± 0.06
	**MBC**	/	171.9 ± 0.03	343 ± 0.02
**Ampicillin**	**MIC**	/	572 ± 0.6	572 ± 0.4
	**MBC**	/	/	/

**Table 3:** Percentage of inhibition of *P. aeruginosa* biofilm formation after the treatment with MIC and 0.5 MIC of compounds, NE—no effect.

Compound	MIC	0.5 MIC
**2**	9.89	15.33
**7**	36.64	NE
**8**	61.34	48.67
**13**	62.69	35.49
**14**	56.74	40.54
**Ampicillin**	71.94	55.42
**Streptomycin**	67.36	30.35

**Table 4:** Results of antifungal potency of the thiazolidinone derivatives (MIC/MFC μM).

Νο.	MIC/MFC	*A.v.*	*T.v.*	*A.o.*	*A.n.*	*P.v.c.*	*P.f.*	*P.o.*	*A.fum.*
**1**	**MIC**	265.8 ± 0.2	132.9 ± 0.08	265.8 ± 0.2	265.8 ± 0.05	132.9 ± 0.2	265.8 ± 0.1	265.8 ± 0.2	265.8 ± 0.02
	**MFC**	531.6 ± 0.3	265.8 ± 0.3	531.6 ± 0.4	531.6 ± 0.03	265.8 ± 0.3	531.6 ± 0.2	531.6 ± 0.3	531.6 ± 0.03
**2**	**MIC**	441.6 ± 0.2	220.8 ± 0.06	220.8 ± 0.5	220.8 ± 0.08	220.8 ± 0.08	220.8 ± 0.03	630.9 ± 0.4	220.8 ± 0.05
	**MFC**	883.2 ± 0.4	441.6 ± 0.2	441.6 ± 0.3	441.6 ± 0.06	441.6 ± 0.3	441.6 ± 0.2	883.2 ± 0.4	441.6 ± 0.06
**3**	**MIC**	94.6 ± 0.4	94.6 ± 0.05	94.6 ± 0.1	189.2 ± 0.09	94.6 ± 0.4	189.2 ± 0.3	94.6 ± 0.4	94.6 ± 0.04
	**MFC**	189.2 ± 0.2	189.2 ± 0.3	189.2 ± 0.4	378.4 ± 0.10	189.2 ± 0.5	378.4 ± 0.3	189.2 ± 0.3	94.6 ± 0.08
**4**	**MIC**	120.8 ± 0.5	60.4 ± 0.02	120.8 ± 0.3	241.6 ± 0.03	120.8 ± 0.05	120.8 ± 0.06	60.4 ± 0.1	120.8 ± 0.05
	**MFC**	241.6 ± 0.3	120.8 ± 0.4	241.6 ± 0.2	483.2 ± 0.06	241.6 ± 0.02	241.6 ± 0.06	120.8 ± 0.02	241.6 ± 0.03
**5**	**MIC**	110.8 ± 0.4	27.7 ± 0.02	55.4 ± 0.3	110.8 ± 0.01	110.8 ± 0.05	110.8 ± 0.08	110.8 ± 0.04	110.8 ± 0.03
	**MFC**	221.6 ± 0.5	55.4 ± 0.03	118 ± 0.4	221.6 ± 0.03	221.6 ± 0.06	221.6 ± 0.02	221.6 ± 0.04	221.6 ± 0.05
**6**	**MIC**	289.0 ± 0.3	144.5 ± 0.05	289.0 ± 0.5	115.6 ± 0.03	115.6 ± 0.06	115.6 ± 0.05	289.0 ± 0.2	231.2 ± 0.002
	**MFC**	578.0 ± 0.4	289.0 ± 0.3	578.0 ± 0.3	231.2 ± 0.05	231.2 ± 0.03	231.2 ± 0.06	289.0 ± 0.4	462.4 ± 0.03
**7**	**MIC**	578.0 ± 0.3	289.0 ± 0.2	289.0 ± 0.5	72.2 ± 0.04	72.2 ± 0.6	34.6 ± 0.08	578.0 ± 0.2	72.2 ± 0.03
	**MFC**	1156 ± 0.3	578.0 ± 0.3	578.0 ± 0.2	144.4 ± 0.06	144.4 ± 0.06	72.2 ± 0.06	1156.0 ± 0.5	144.4 ± 0.04
**8**	**MIC**	156.7 ± 0.6	156.7 ± 0.08	313.4 ± 0.4	501.5 ± 0.08	501.5 ± 0.5	501.5 ± 0.2	313.4 ± 0.3	501.5 ± 0.06
	**MFC**	313.4 ± 0.3	313.4 ± 0.3	626.9 ± 0.4	1003.0 ± 1	1003.0 ± 0.6	1003.0 ± 0.3	626.9 ± 0.3	1003.0 ± 1
**9**	**MIC**	250.7 ± 0.5	141.0 ± 0.04	282.1 ± 0.4	250.8 ± 0.04	250.8 ± 0.08	250.8 ± 0.08	501.5 ± 0.3	250.8 ± 0.04
	**MFC**	501.5 ± 0.3	282.1 ± 0.05	564.2 ± 0.1	501.6 ± 0.03	501.6 ± 0.3	501.6 ± 0.3	1003.1 ± 0.2	501.6 ± 0.08
**10**	**MIC**	119.2 ± 0.2	59.6 ± 0.02	119.2 ± 0.6	119.2 ± 0.05	59.6 ± 0.02	59.6 ± 0.03	59.6 ± 0.05	59.6 ± 0.02
	**MFC**	238.4 ± 0.2	119.2 ± 0.05	238.4 ± 0.5	238.4 ± 0.08	119.2 ± 0.03	119.2 ± 0.01	119.2 ± 0.03	119.2 ± 0.06
**11**	**MIC**	432.4 ± 0.4	108.1 ± 0.06	216.2 ± 0.4	378.4 ± 0.04	189.2 ± 0.05	189.2 ± 0.06	432.4 ± 0.5	378.4 ± 0.06
	**MFC**	864.8 ± 0.5	216.2 ± 0.05	432.4 ± 0.4	756.7 ± 0.06	378.4 ± 0.3	378.4 ± 0.08	864.8 ± 0.5	756.7 ± 0.08
**12**	**MIC**	135.1 ± 0.3	135.1 ± 0.06	270.2 ± 0.3	135.1 ± 0.06	135.1 ± 0.06	270.2 ± 0.06	135.1 ± 0.3	270.2 ± 0.05
	**MFC**	270.2 ± 0.2	270.2 ± 0.2	540.4 ± 0.2	270.2 ± 0.03	270.2 ± 0.3	540.4 ± 0.3	270.2 ± 0.2	540.4 ± 0.03
**13**	**MIC**	81.0 ± 0.2	81.0 ± 0.06	162.0 ± 0.3	162.0 ± 0.06	81.0 ± 0.04	81.0 ± 0.04	81.0 ± 0.05	81.0 ± 0.01
	**MFC**	162.0 ± 0.2	162.0 ± 0.04	324.0 ± 0.5	324.0 ± 0.08	162.0 ± 0.04	162.0 ± 0.04	162.0 ± 0.3	162.0 ± 0.05
**14**	**MIC**	78.9 ± 0.4	78.9 ± 0.03	157.9 ± 0.2	157.9 ± 0.04	78.9 ± 0.02	78.9 ± 0.05	78.9 ± 0.3	157.9 ± 0.03
	**MFC**	157.9 ± 0.3	157.9 ± 0.2	315.8 ± 0.1	315.8 ± 0.04	157.9 ± 0.08	157.9 ± 0.01	157.9 ± 0.4	315.8 ± 0.06
**15**	**MIC**	342.2 ± 0.2	171.1 ± 0.4	342.2 ± 0.5	342.2 ± 0.08	342.2 ± 0.02	342.2 ± 0.03	342.2 ± 0.03	342.2 ± 0.04
	**MFC**	684.4 ± 0.3	342.2 ± 0.5	684.4 ± 0.1	684.4 ± 0.03	684.4 ± 0.06	684.4 ± 0.02	684.4 ± 0.5	684.4 ± 0.05
**Bifonazole**	**MIC**	480.0 ± 0.3	640.0 ± 0.1	480.0 ± 0.1	480.0 ± 0.10	480.0 ± 0.2	640 ± 0.2	480 ± 0.3	480 ± 0.06
	**MFC**	640.0 ± 0.5	800 ± 0.3	800.0 ± 0.4	640.0 ± 0.08	640.0 ± 0.2	800 ± 0.2	640 ± 0.4	640 ± 0.03
**Ketoconazole**	**MIC**	2850 ± 0.3	4750.0 ± 0.3	380.0 ± 0.2	380.0 ± 0.06	380.0 ± 0.3	380 ± 0.3	3800 ± 0.3	380 ± 0.06
	**MFC**	3800 ± 0,06	5700.0 ± 0.2	950.0 ± 0.5	950.0 ± 0.10	950.0 ± 0.3	950 ± 0.4	3800 ± 0.5	950 ± 0.08

*A.v.—A. versicolor, T.v.—T. viride, A.o.—A. ochraceus, A.n.—A. niger, P.v.c.—P. cyclopium var verrucosum, P.f.—P. funiculosum, P.o.—P. ochrochloron, A.fum.—A. fumigatus*.

**Table 5:** Significant physicochemical parameters for the passage of biological membranes.

No.	TPSA (A^2^)	Μ.Β.	nOHNH	nON	Violations	cLogP
**1**	58.12	301.4	1	4	0	3.67
**2**	78.35	317.39	2	5	0	3.28
**3**	78.35	317.39	2	5	0	3.28
**4**	67.35	331.42	1	5	0	3.54
**5**	76.59	261.45	1	6	0	3.42
**6**	103.94	346.39	1	7	0	3.88
**7**	103.94	346.39	1	7	0	3.88
**8**	58.12	319.39	1	4	0	3.83
**9**	58.12	319.39	1	4	0	3.83
**10**	58.12	335.84	1	4	0	4.23
**11**	58.12	270.29	1	4	0	4.78
**12**	58.12	370.29	1	4	0	4.78
**13**	58.12	370.29	1	4	0	4.78
**14**	58.12	380.29	1	4	0	4.50
**15**	58.12	380.29	1	4	0	4.50

## Data Availability

The data presented in this study are available on request from the corresponding author.

## Supplementary Materials

The following are available online at https://www.mdpi.com/2079-6382/10/3/309/s1, Table S1: Predict toxicity of compounds with OpenTox and CBLIGAND title, 1H-13C-NMR spectra mass spectras, docking studies. Table S2: Computed docking scores and relevant amino acids interacting involving E. Coli-Mur B. Table S3: Molecular docking free binding energies (kcal/mol) on antifungal targets.
