# Supplementary material for: 5-Benzyliden-2-(5-methylthiazol-2-ylimino)thiazolidin-4-ones as Antimicrobial Agents. Design, Synthesis, Biological Evaluation and Molecular Docking Studies
Source: Antibiotics (Basel). 2021 Mar 17;10(3):309. doi: 10.3390/antibiotics10030309 (PMC8002837; doi:10.3390/antibiotics10030309)
Supplement: Supplementary file 1 [file antibiotics-10-00309-s001.zip › antibiotics-1060612-supplementary/Supplementary files/compounds.docx]

| **No** | **R** | **No** | **R** |
| --- | --- | --- | --- |
| **1** | H | **10** | 4-F |
| **2** | 4-OH | **11** | 2,6-di-F |
| **3** | 4-OMe | **12** | 4-Br |
| **4** | 4-OH, 3-OMe | **13** | 3-Cl |
| **5** | 4-Me | **14** | 4-Cl |
| **6** | 2-NO_2_ | **15** | 2,3-di-Cl |
| **7** | 3-NO_2_ | **16** | 2,4-di-Cl |
| **8** | 4-NO_2_ | **17** | 2,6-di-Cl |
| **9** | N(CH_3_)_2_ |  |  |

| **No** | **MW** | **No** | **MW** |
| --- | --- | --- | --- |
| **1** | **301** | **9** | **316** |
| **2** | **317** | **10** | **335,5** |
| **3** | **307** | **11** | **370** |
| **4** | **331** | **12** | **370** |
| **5** | **361** | **13** | **370** |
| **6** | **346** | **14** | **380** |
| **7** | **346** | **15** | **380** |
| **8** | **319** |  |  |
