# Supplementary material for: 5-Benzyliden-2-(5-methylthiazol-2-ylimino)thiazolidin-4-ones as Antimicrobial Agents. Design, Synthesis, Biological Evaluation and Molecular Docking Studies
Source: Antibiotics (Basel). 2021 Mar 17;10(3):309. doi: 10.3390/antibiotics10030309 (PMC8002837; doi:10.3390/antibiotics10030309)
Supplement: Supplementary file 1 [file antibiotics-10-00309-s001.zip › antibiotics-1060612-supplementary/Supplementary files/Docking studies.docx]

5-Benzyliden-2-(5-methylthiazol-2-ylimino)thiazolidin-4-ones as antimicrobial agents. Design, synthesis, biological evaluation and molecular docking studies

Michelyne Haroun^1^, Christophe Tratrat^1^, Aggeliki Kolokotroni^2^ Anthi Petrou^2^, Athina Geronikaki^2^*, Marija Ivanov^3^, Marina Kostic^3^, Marina Sokovic^3^, Alejandro Carazo^4^, Přemysl Mladěnka,^4^ Sree Harsha^1^, Katharigatta N. Venugopala^1,5^, Anroop B. Nair^1^, Heba S. Elsewedya^1^

*2.4. Docking Studies*

2.4.1. Docking in *E. coli*-MurB

It was previously demonstrated that thiazolidinone derivatives act as MurB inhibitors [48-50]. MurB is an enzyme belonging to the superfamily of flavoproteins and plays a key role in cell wall biosynthesis as it participates in the second stage of synthesis of peptidoglycan, which is a crucial component of bacterial cell wall. In particular, it catalyzes the final UDP-N-acetylmuramic acid (UDPMurNAc) formation step by reducing the NADPH-dependent enol pyruvate. Taking this into account and looking to study the mode of action of our compounds, a theoretical study of their binding to *E. coli*-MurB enzyme active site (PDB:2Q85) was accomplished. Results are presented in Table S2 .

**Table S2.** Computed docking scores and relevant amino acids interacting involving *E. Coli*-Mur B.

| **No** | **Binding affinity** | **Free binding energy (kcal/mol)** | **I-H** | **Residues involved in Hydrogen Bonds** | **Attractive charge, Pi-Anion** | **Alkyl, Pi-Alkyl interactions** | **Halogen Bond** | **Hydrophobic**  **interactions** |
| --- | --- | --- | --- | --- | --- | --- | --- | --- |
| **1** | -13.71 | -6.54 | - | - | - | Ile109, Ala123 | - | Asn50, Ile121, Gly122, Tyr189, Arg326 |
| **2** | -31.55 | -10.37 | 2 | Arg213, Ser228 | - | Ile109, Ile121, Pro218, Leu217, Val290 | - | Asn50, Pro110, Ser115, Ile118, Ile121, Ala123, Arg158, Pro220, Gly227, Gln287, Leu289, Glu324, Arg326 |
| **3** | -24.37 | -8.19 | 1 | Arg213 | - | Ile109, Pro110, Leu217 | - | Asn50, Gln119, Ile121, Gly122, Tyr189, Arg326 |
| **4** | -26.22 | -9.11 | 2 | Tyr124, Arg158 | - | Ile109, Pro110, Ala123 | - | Asn50, Ile118, Gly227, Ser228, Gln287, Glu324 |
| **5** | -20.79 | -7.11 | 1 | Gly122 | - | Pro110 |  | Asn50, Arg158, Leu289 |
| **6** | -25.47 | -8.56 | 1 | Arg158 | - | Pro218, Leu217 | - | Asn50, Pro110, Ile118, Ala123, Ser228, Leu289 |
| **7** | -39.46 | -11.56 | 4 | Tyr124, Arg158, Arg213, Ser228 | Glu324 | Ala123 | - | Asn50, Ile109, Gln119, Ile121, Gly122, Gly125, Tyr189, Leu217, Gly227, Arg326 |
| **8** | -27.48 | -9.78 | 2 | Tyr124, Arg158 | - | Ile109, Pro110, Ile121 | - | Asn50, Ser115, Gln119, Ala123, Pro220, Gly227, Gln287, Leu289, Arg326 |
| **9** | -9.94 | -6.22 | - | - | Arg213 | Ile109, Pro110, Ala123, Leu217 | Ser115 | - |
| **10** | -24.75 | -8.02 | 1 | Arg158 | - | Pro218, Leu217 | - | Asn50, Ile118, Ala123, Arg158, Ser228, Leu289 |
| **11** | -21.64 | -7.42 | 1 | Ser228 | - | Ile109, Leu217 | - | Ala123, Gly227, Gln287, Leu289, Arg326 |
| **12** | -19.48 | -7.00 | 1 | Ser228 | - | , Pro110, Ala123, Leu217 | - | Asn50, Ile109, Ile121, Gly122, Tyr189, Leu217 |
| **13** | -26.13 | -9.14 | 2 | Arg213, Arg326 | - | Ile109, Ile121, Leu217 | - | Asn50, Pro110, Ile118, Ala123, Arg158, Ser228, Leu289, Arg326 |
| **14** | -29.31 | -10.11 | 2 | Ser228, Arg326 | - | Pro218, Leu217 | - | Asn50, Pro110, Ile118, Ile121, Arg158, Pro220, Gly227, Gln287, Glu324, Arg326 |
| **15** | -10.86 | -6.28 | - | - | - | Ile109, Leu217 | - | Asn50, Gly125, Arg326 |

Docking studies displayed the lowest binding energy (-11.56) of the most potent derivative (**7**). Ligand-enzyme interactions exhibited 4 H-bonds: among the oxygen of NO_2_ substituent and the hydrogen atoms localized on the side chain of the amino acids Arg158 and Tyr124 (2.40 and 2.34 respectively), a hydrogen bond between the thiazolidinone O and H atoms of NH of the Ser228 amino acid part (2,28) and one more between the sulfur of the thiazolidinone and the hydrogen of the NH group of the amino acid Arg213 (2,74) [48]. The compound is directed to deeply enter *E. coli* MurB enzyme active site, to a "channel" formed by the amino acids Gly125, Gly227, Glu324, Ile121, Asn50, Gly122, Ile209 and Arg326. The hydrogen bonds in combination with the hydrophobic and aromatic interactions stabilize the binding of the compound to the active center of the enzyme (Figure 1). It is worthy to highlight that the hydrogen bond with the residue Ser228 is crucial for the inhibitory action of this compound, because this residue takes part in the proton transfer at the second stage of peptidoglycan synthesis [51]. Hydrogen bond interactions with the residue Ser228 were also observed for the compounds **2**, **11, 12** and **14** which exhibited very low free binding energies (table S2).


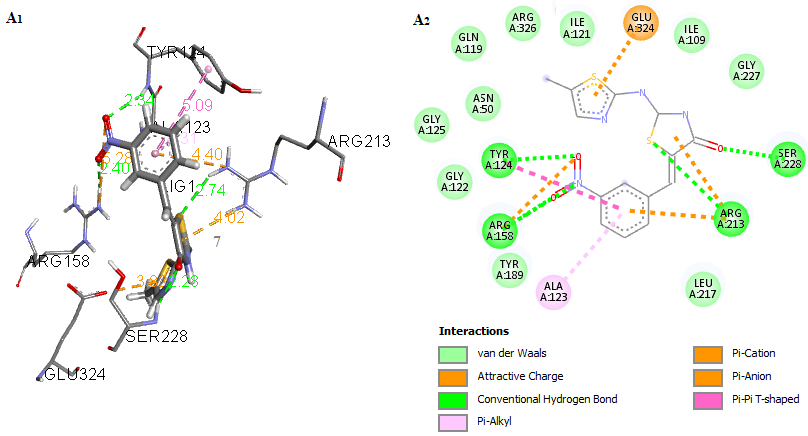


**Figure 1**. The binding mode of compound **7** (-11.56 minimum energy) in the enzyme active side of *E. coli* MurB (PBD: 2Q85). A green dotted line shows the hydrogen bonds, a yellow dotted and purple-colored line represent hydrophobic and aromatic interactions respectively.

Compound **2** exhibited equally good inhibitory activity on the enzyme with the minimum binding free energy (-10.37). It forms 2 hydrogen bonds between the thiazolidinone oxygen and hydrogen of the Arg213 amino acid side chain NH group (2.03A) and between the OH and the oxygen of the amino acid Ser228 (2.37A). It also shows a number of hydrophobic interactions with the amino acids Ile121, Arg158, Gln287, Gly227, Asn225, Val290, Asn50, Pro218, Aeg326 and Leu217 (Figure 2).

Compound **9** exhibited the smallest effect on the enzyme with minimal binding energy (-6.22) and with only few hydrophobic interactions with the amino acids Glu227, ley217, Ala123, Arg213, Pro110 and Ile109 (Figure 3). These findings are in agreement with experimental results.


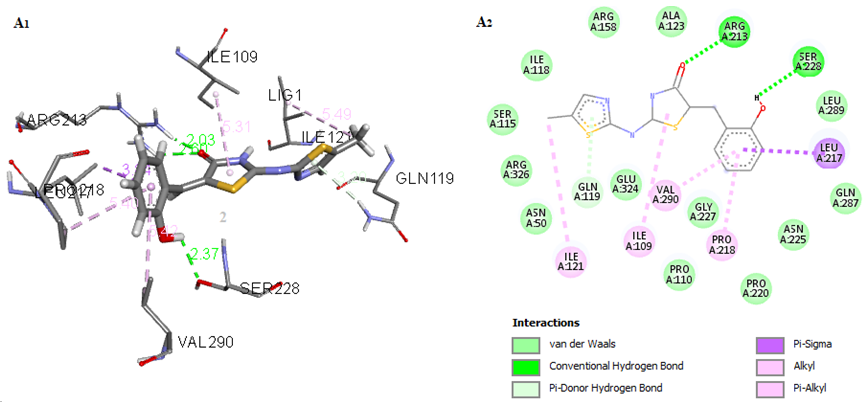


**Figure 2.** Representation of compound **2** binding (-10.37miniest energy) and *E. coli* MurB (PBD: 2Q85) at the enzyme active site. A green dotted line shows the hydrogen bonds, a yellow and purple-colored dotted lines represent hydrophobic and aromatic interactions respectively.


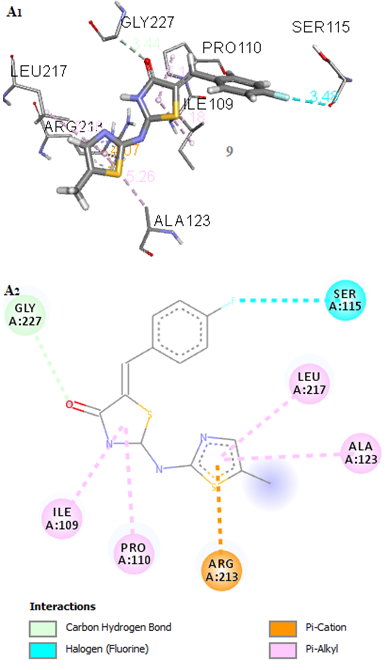


**Figure 3.** Binding of compound **9** (-6.22 minimum energy) and *E. coli* MurB (PBD: 2Q85) to the enzyme active site. A green line dotted line shows the hydrogen bonds, a yellow and purple-colored dotted line represent hydrophobic and aromatic interactions respectively.

2.4.2. Docking in antifungal targets

Prepared derivatives **1**-**15** and standard drug were subjected to docking with lanosterol 14α-demethylase from pathogenic yeast *C*. *albicans* (CYP51_Ca_) and dihydrofolate reductase to predict the probable mechanism of action using PDB:5V5Z and PDB:4HOF) respectively. The docking scores disclosed that CYP51_Ca_ was the most adequate to antifungal potency (Table S3).

**Table S3** Molecular docking free binding energies (kcal/mol) on antifungal targets

| **No** | **Est. binding energy(kcal/mol)** | | **I-H** | **Residues involved in Hydrogen Bonds** | **Aromatic-positive ionizable interactions** | **Hydrophobic interactions** | **Interactions with**  **HEM601**  **CYP51_Ca_** |
| --- | --- | --- | --- | --- | --- | --- | --- |
|  | **Dihydrofolate reductase**  **(PDB: 4HOF)** | **CYP51_Ca_**  **(PDB: 5V5Z)** |  |  |  |  |  |
| **1** | -5.26 | -8.72 | 2 | Tyr118, Tyr132 | - | Ile131, Leu300, Leu376, Met508 | **Hydrophobic** |
| **2** | -2.15 | -8.55 | 1 | Tyr132 | - | Tyr118, Tyr132, Leu376, Met508 | **Hydrophobic** |
| **3** | -3.02 | -8.97 | 2 | Tyr64, His377 | Tyr188 | Tyr118, Ile131, Tyr132, Leu300, Leu376, Met508 | **Hydrophobic** |
| **4** | -2.55 | -8.77 | 2 | Tyr118, Tyr132 | - | Tyr118, Ile131, Tyr132, Leu376, Met508 | **Hydrophobic** |
| **5** | -1.29 | -9.92 | 1 | Tyr118, His377 | Tyr188 | Tyr122, Ile131, Tyr132, Leu376, Met508 | **Hydrophobic** |
| **6** | -3.44 | -8.81 | 2 | Tyr64, Tyr118 | - | Ile131, Tyr132, Leu300, Leu376, Met508 | **Hydrophobic** |
| **7** | -271 | -8.70 | 2 | Tyr118, Tyr132 | - | Tyr64, Ile131, Tyr132, His377, Met508 | **Hydrophobic** |
| **8** | -2.66 | -7.35 | 1 | His377 | - | Tyr122, Leu376, Met508 | **Hydrophobic** |
| **9** | -1.04 | -8.73 | 2 | Tyr118, His377 | - | Tyr118, Ile131, Il;e304, Leu376, Met508 | **Hydrophobic** |
| **10** | -5.17 | -10.13 | 2 | Met508, His377 | - | Tyr118, Tyr122, Ile131, Tyr132, Tyr305, Leu376, Val510 | **Hydrophobic** |
| **11** | -2.11 | -8.42 | 1 | Tyr118 | - | Tyr122, Tyr132, Leu376 | **Hydrophobic** |
| **12** | -1.67 | -8.86 | 2 | Tyr118, Met508 | Tyr132 | Ile131, Ile304, Leu376, Met508 | **Hydrophobic** |
| **13** | -3.57 | -9.55 | 2 | Tyr64, Tyr118 | Tyr188 | Tyr118, Ile131, Tyr132, Leu376, Met508, Val510 | **Hydrophobic** |
| **14** | -1.98 | -9.21 | 2 | Tyr118, Met508 | Tyr188 | Ile131, Tyr132, Leu376 | **Hydrophobic** |
| **15** | -4.17 | -7.28 | 1 | His377 | - | Tyr122, Tyr132, Met508 | **Hydrophobic** |
| **Ket.** | - | -8.23 | 1 | Tyr64 | - | Tyr118, Ile131, Tyr132, Ile304, Leu300, Leu376, Met508 | **Ionizable, Hydrophobic** |

Docking assessment highlighted the evidence that all the prepared derivatives may bind to CYP51_Ca_ in an analogous mode to that of standard antifungal drug ketoconazole. Derivative **10** is situated inside the enzyme by the side of heme group, interacting hydrophobically with it. Furthermore, compound **10** forms two H-bonds. The first H-bond is formed between the nitrogen of thiazolidinone moiety and the side-chain hydrogen of Met508, and the second one, between nitrogen of thiazole moiety and the hydrogen located on His377 side chain. Hydrophobic interactions were detected between residues Thr122, Tyr188, Leu376, Tyr132 and Ile131 and the benzene ring of the compound **10**, also between Tyr505, Val510, His377 and the thiazole ring of the compound. Additionally, compound **10** interacts hydrophobically throughout its benzene ring with the heme group of the enzyme (figure 4). Despite positive ionizable interactions between benzene skeleton of ketoconazole and the heme group were recorded (figure 4 and 5), compound 10 forms strongest ligand-enzyme complex due to hydrogen bond interactions. This is probably the reason of increased antifungal activity of compound **10** compared with the standard (ketoconazole).


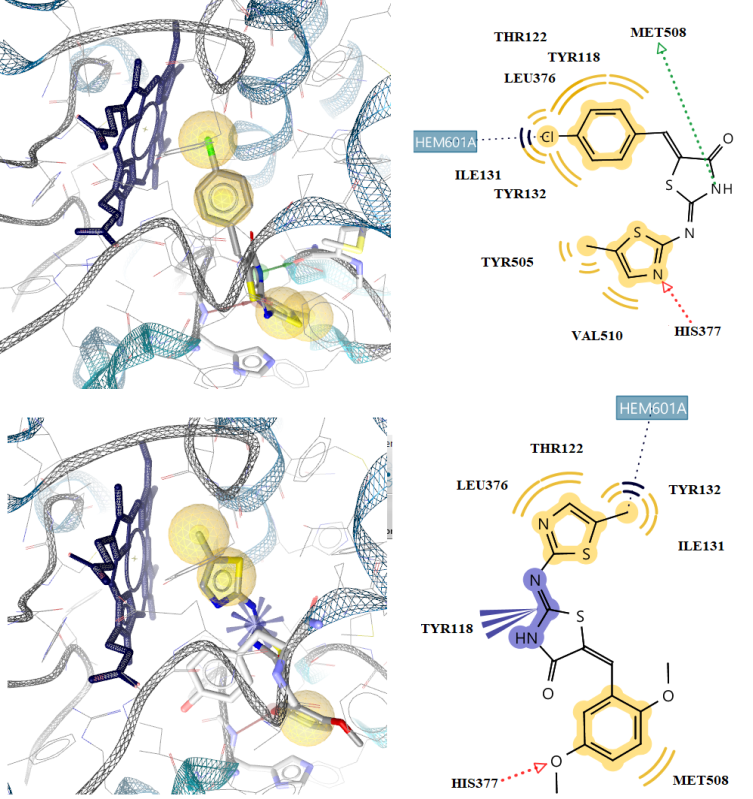


**Figure 4.** Docking of the most active compounds **10 (**up) and **5** (down) in CYP51_Ca_ receptor.

**
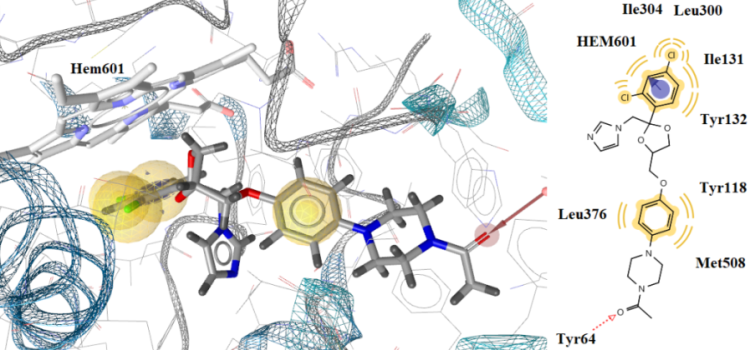
**

**Figure 5.** Docking of ketoconazole on CYP51_Ca_ receptor.

*3.4. Molecular Modeling Studies*

Docking studies were performed after the biological evaluation in an effort to predict the mechanism of action of the compounds. All calculations were performed utilizing Autodock 4.2 program [64]. ΔG (binding free energy) of selected structures was calculated using the above-mentioned molecular binding program. To prepare proteins, all water molecules were eliminated and polar hydrogens added, while for preparation of the inhibitors, the charges were added and the rotatable bonds determined. Grid maps have been calculated utilizing Autogrid algorithm and must contain the area to be connected. Autogrid Box was computed by the X-, Y- and Z-coordinates for each enzyme. Three-dimensional structures of all compounds were constructed using Chem3Dultra 12.0 software (Chemical Structure Drawing Standard; Perkin Elmer Informatics, Waltham, MA, USA).  For the present system, the Lamarckian Genetic Algorithm was applied for minimization and the following settings were used: initial population: 300, 2,500,000 maximum energy ratings and 27,000 as maximum generation. The pitch was 1,0 Å while the quaternion and pivot angle were set to 5,0 degrees. For each compound, 200 configurations were produced. The results from the Autodock calculations were grouped using a RMSD deviation value of 1.5 Å, while the lowest-energy configuration of the largest population group was chosen as the most likely tethering configuration. The discovery studio 2017 R2 silent and LigandScout were used to display the results and process the configurations with the highest tie rating [65].

3.4.1. Docking studies for prediction of the mechanism of antibacterial activity

Since, thiazolidinone derivatives have been found to act as MurB inhibitors [48,49], the structure 2Q85 of *E. coli* MurB with its inhibitor, (5Z)-3-(4-chlorophenyl)-4-hydroxy-5-(naphthalene -1-ylmethylidene) was chosen for docking studies. The docking box was centered at x=4.97, y=−8.78 and z=10.62 with a target box of 50 x 50 x 50 Å.

3.4.2. Docking studies for prediction of the mechanism of anti-fungal activity

A common anti-fungal drug target is the enzyme CYP51 14α-lanosterol demethylase that has been found to be inhibited by thiazolidinone derivatives [48]. So, lanosterol 14a-demethylase, CYP51, enzyme from *C. albicans* with PDB I.D. 5V5Z, was chosen for docking studies for the antifungal activity of the compounds. The docking box was centered on the heme molecule, at the active center of the enzyme, with coordinates x=−47.731, y=−13.422, z=22.982 with a target box of 50×50×50Å. As a first step of docking studies, the initial inhibitor 2-[(2R)-butan-2-yl]-4-{4-[4-(4- {[(2R,4S)-2-(2,4-dichlorophenyl)-2-(1H-1,2,4-triazol-1-ylmethyl)-1,3-dioxolan-4-yl]methoxy}phenyl)piperazin-1-yl]phenyl}-2,4-dihydro-3H-1,2,4-triazol-3-one, was removed and docked to the prepared enzyme for verification of the method (Figure 6) with RMSD value 0.855Å. Furthermore, the reference drug ketoconazole was also docked into the active site of 5V5Z structure.


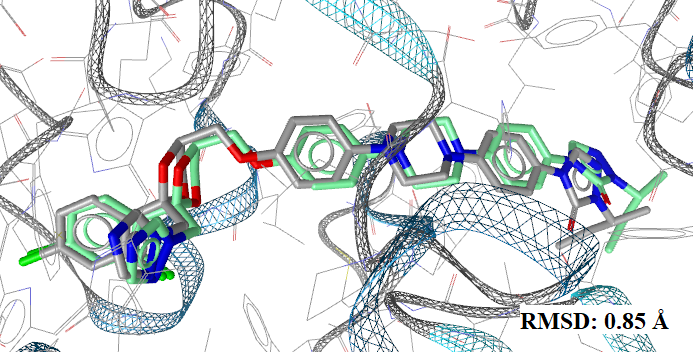


**Figure.6** Docking of the initial inhibitor to the 5V5Z structure.
