# Supplementary material for: 5-Benzyliden-2-(5-methylthiazol-2-ylimino)thiazolidin-4-ones as Antimicrobial Agents. Design, Synthesis, Biological Evaluation and Molecular Docking Studies
Source: Antibiotics (Basel). 2021 Mar 17;10(3):309. doi: 10.3390/antibiotics10030309 (PMC8002837; doi:10.3390/antibiotics10030309)
Supplement: Supplementary file 1 [file antibiotics-10-00309-s001.zip › antibiotics-1060612-supplementary/Supplementary files/MS spectra.docx]

**LC-MS conditions**

For LC/MS analyses, a Shimadzu LC/MS 2020 single quadrupole mass spectrometer with an electrospray ion source (ESI) was used. A nitrogen gas generator N2LCMS (Nitrogen Generator, Claind) was employed throughout in this study. The temperature of the dessolvation line and heat block were set at 250 and 200 °C, respectively. The N_2_ nebulizer gas flow was maintained at 1.5 L min^-1^ and the drying gas flow was set at 15 L min^-1^, while the interface voltage was set at 4.5 kV in positive or negative mode. The sample injection volume was 5 μL in all cases. The carrier was a mixture of 0.1% aqueous formic acid/methanol, 50/50 v/v. The flow rate was set at 0.5 mL min^-1^.

MS scan: 100 – 500 m/z

Sample concentration: 100 μg mL^-1^ in MeOH

**Sample 1**

**Sample 2**

**Sample 3**

125.0

150.0

175.0

200.0

225.0

250.0

275.0

300.0

325.0

350.0

375.0

400.0

425.0

450.0

475.0

m/z

0.0

1.0

2.0

3.0

4.0

5.0

Inten.

(x100,000)

398

265

425

337

298

173

369

233

321

118

353

155

139

308

**Sample 4**

**Sample 5**

**Sample 6**

**(+)ve**

**(-)ve**

**Sample 7**

**(+)ve**

**(-)ve**

**Sample 8**

**Spectra 9**

**Sample 10**

125.0

150.0

175.0

200.0

225.0

250.0

275.0

300.0

325.0

350.0

375.0

400.0

425.0

450.0

475.0

m/z

0.0

1.0

2.0

3.0

4.0

5.0

Inten.

(x100,000)

217

398

252

265

475

425

337

298

173

369

233

321

118

353

155

139

335

**Sample 11**

**Zoomed MS spectra**

**Sample 12**

**Zoomed MS spectra**

**Sample 13**

**Zoomed MS spectra**

**Sample 14**

**Zoomed MS spectra**

**Sample 15**

**Zoomed MS spectra**
