# Supplementary material for: 5-Benzyliden-2-(5-methylthiazol-2-ylimino)thiazolidin-4-ones as Antimicrobial Agents. Design, Synthesis, Biological Evaluation and Molecular Docking Studies
Source: Antibiotics (Basel). 2021 Mar 17;10(3):309. doi: 10.3390/antibiotics10030309 (PMC8002837; doi:10.3390/antibiotics10030309)
Supplement: Supplementary file 1 [file antibiotics-10-00309-s001.zip › antibiotics-1060612-supplementary/Supplementary files/Table S1.docx]

**Table S1**. Predict toxicity of compounds with OpenTox and CBLIGAND.

| **Α/Α** | **R** | **Toxicity for eyes** | **Toxicity for skin** | **Carcinogenesis (rats)** | **Carcinogenesis (mice)** | **Mutagenicity**  **(*Salmonella typhimurium*)** |
| --- | --- | --- | --- | --- | --- | --- |
| **1** | **H** | Not irritating | Not irritating | Not expected | Not expected | Not expected |
| **2** | **2-OH** | Not irritating | Not irritating | Not expected | Not expected | Not expected |
| **3** | **4-OH** | Not irritating | Not irritating | Not expected | Not expected | Not expected |
| **4** | **4-OCH_3_** | Not irritating | Not irritating | Not expected | Not expected | Not expected |
| **5** | **2,5-di-OCH_3_** | Not irritating | Not irritating | Not expected | Not expected | Not expected |
| **6** | **2-NO_2_** | Not irritating | Not irritating | Not expected | Not expected | Not expected |
| **7** | **3-NO_2_** | Not irritating | Not irritating | Not expected | Not expected | Not expected |
| **8** | **3-F** | Not irritating | Not irritating | Not expected | Not expected | Not expected |
| **9** | **4-F** | Not irritating | Not irritating | Not expected | Not expected | Not expected |
| **10** | **4-Cl** | Not irritating | Not irritating | Not expected | Not expected | Not expected |
| **11** | **2,3-di-Cl** | Not irritating | Not irritating | Not expected | Not expected | Not expected |
| **12** | **2,4-di-Cl** | Not irritating | Not irritating | Not expected | Not expected | Not expected |
| **13** | **2,6-di-Cl** | Not irritating | Not irritating | Not expected | Not expected | Not expected |
| **14** | **3-Br** | Not irritating | Not irritating | Not expected | Not expected | Not expected |
| **15** | **4-Br** | Not irritating | Not irritating | Not expected | Not expected | Not expected |
