# Supplementary figures and images for: 5-Benzyliden-2-(5-methylthiazol-2-ylimino)thiazolidin-4-ones as Antimicrobial Agents. Design, Synthesis, Biological Evaluation and Molecular Docking Studies
Source: Antibiotics (Basel). 2021 Mar 17;10(3):309. doi: 10.3390/antibiotics10030309 (PMC8002837; doi:10.3390/antibiotics10030309)

**-1-**

**
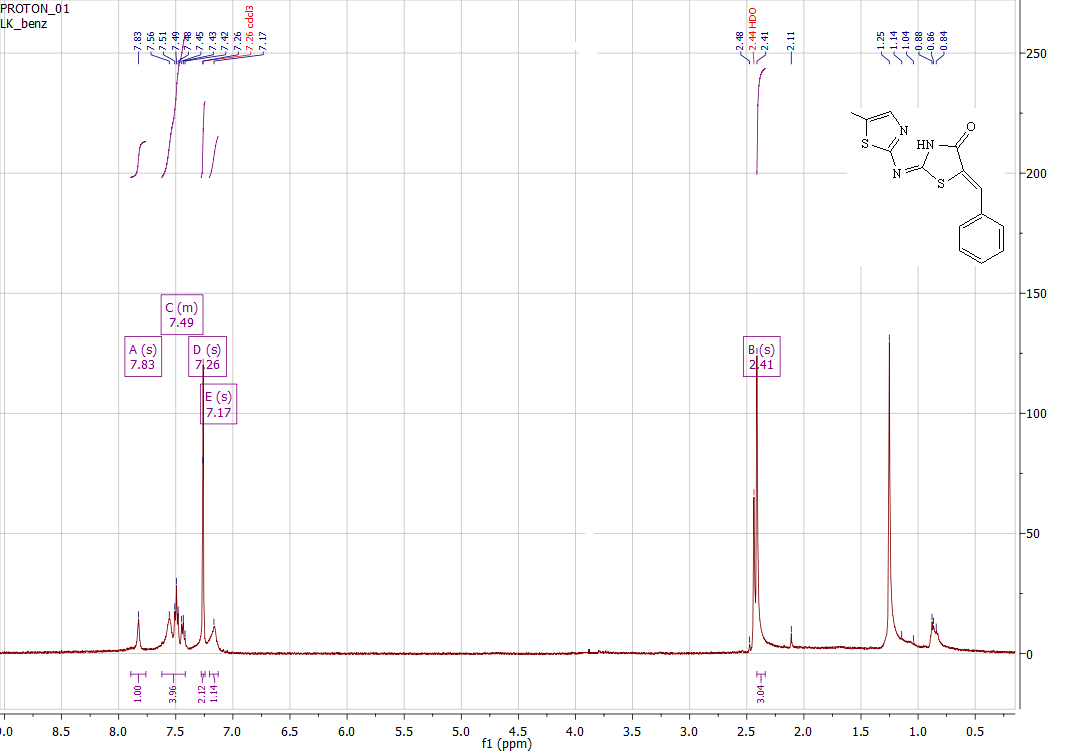
**

**
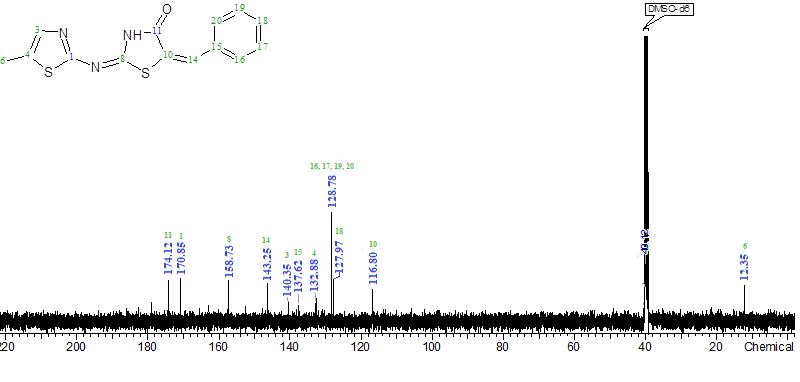
**

**-2-**

**
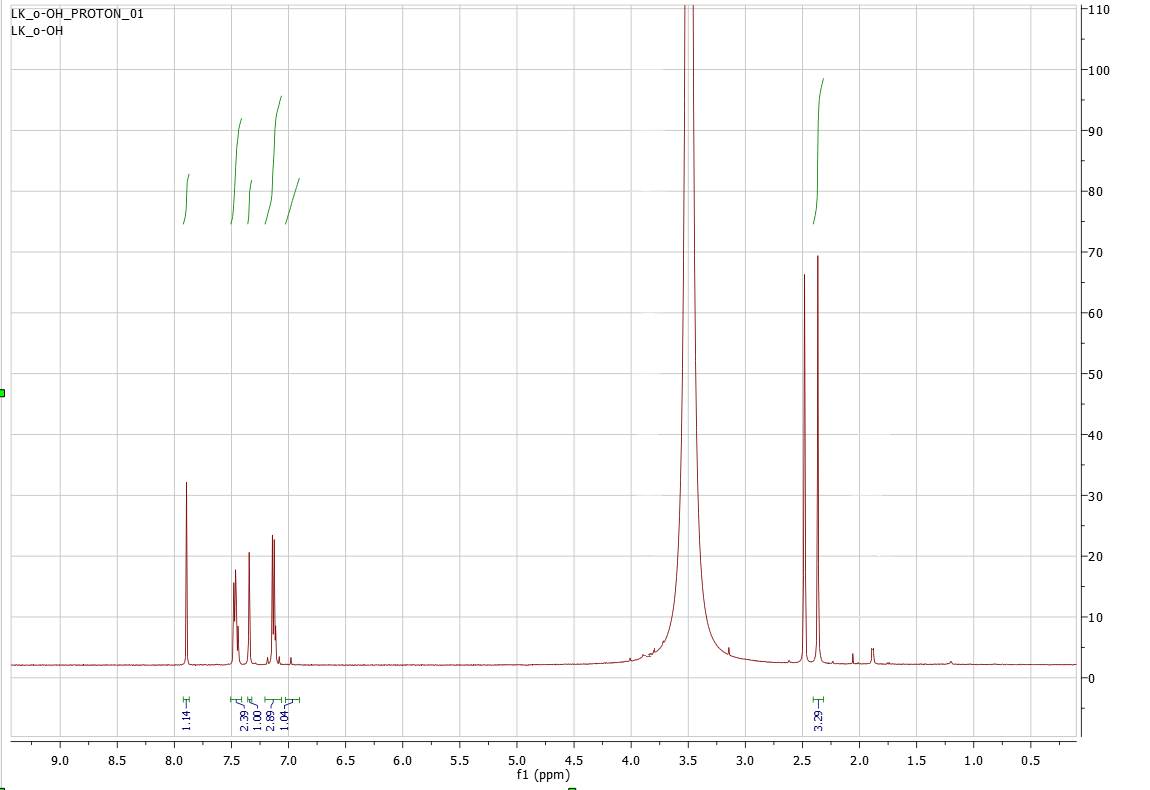
**

**
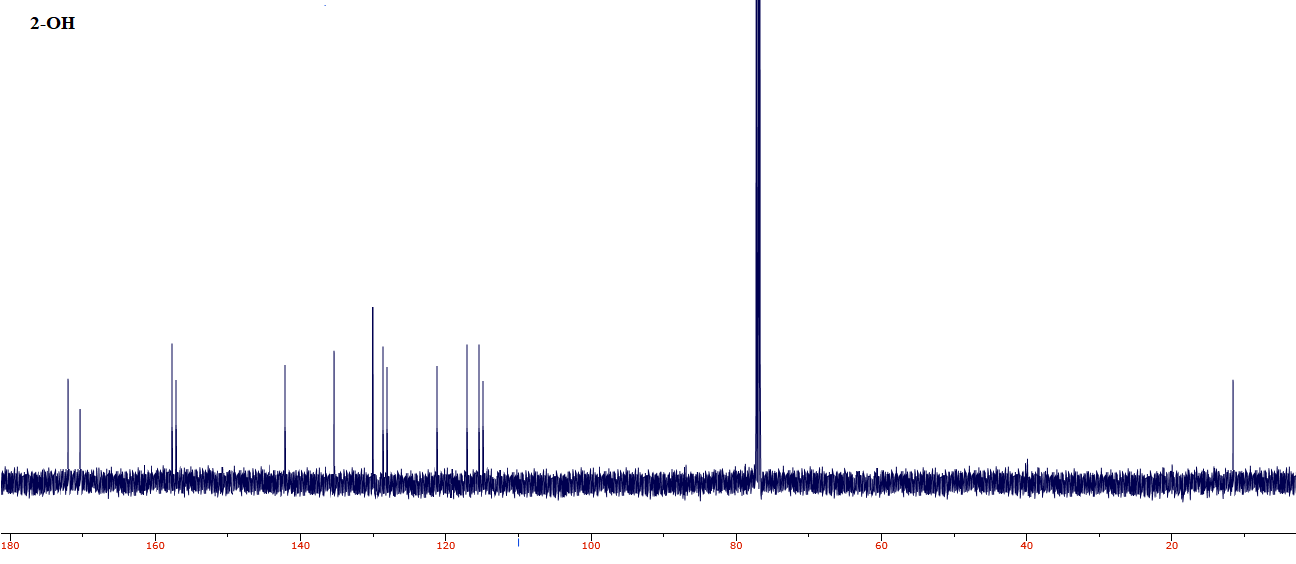
**

**-3-**

**
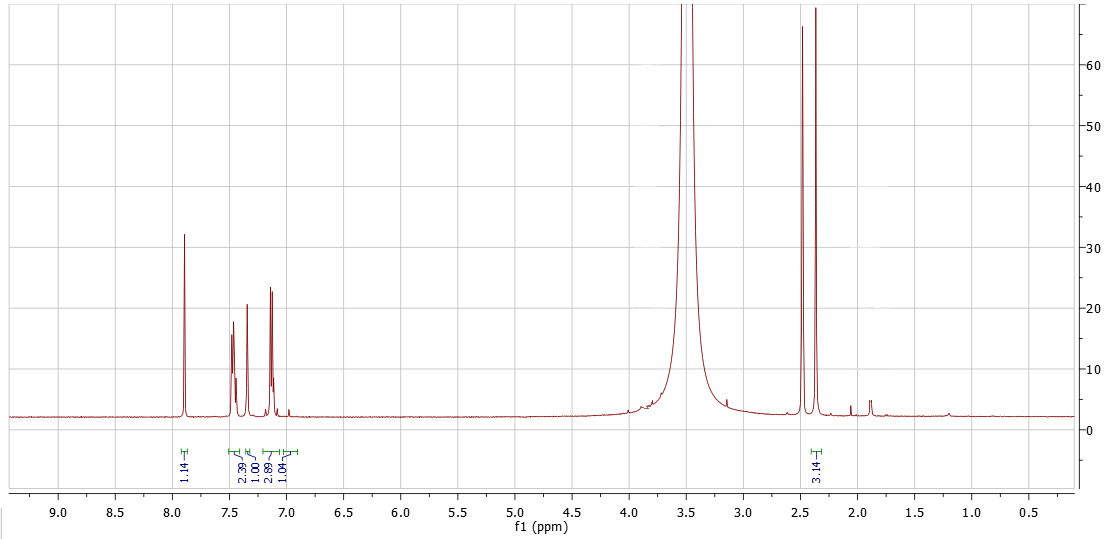
**

**
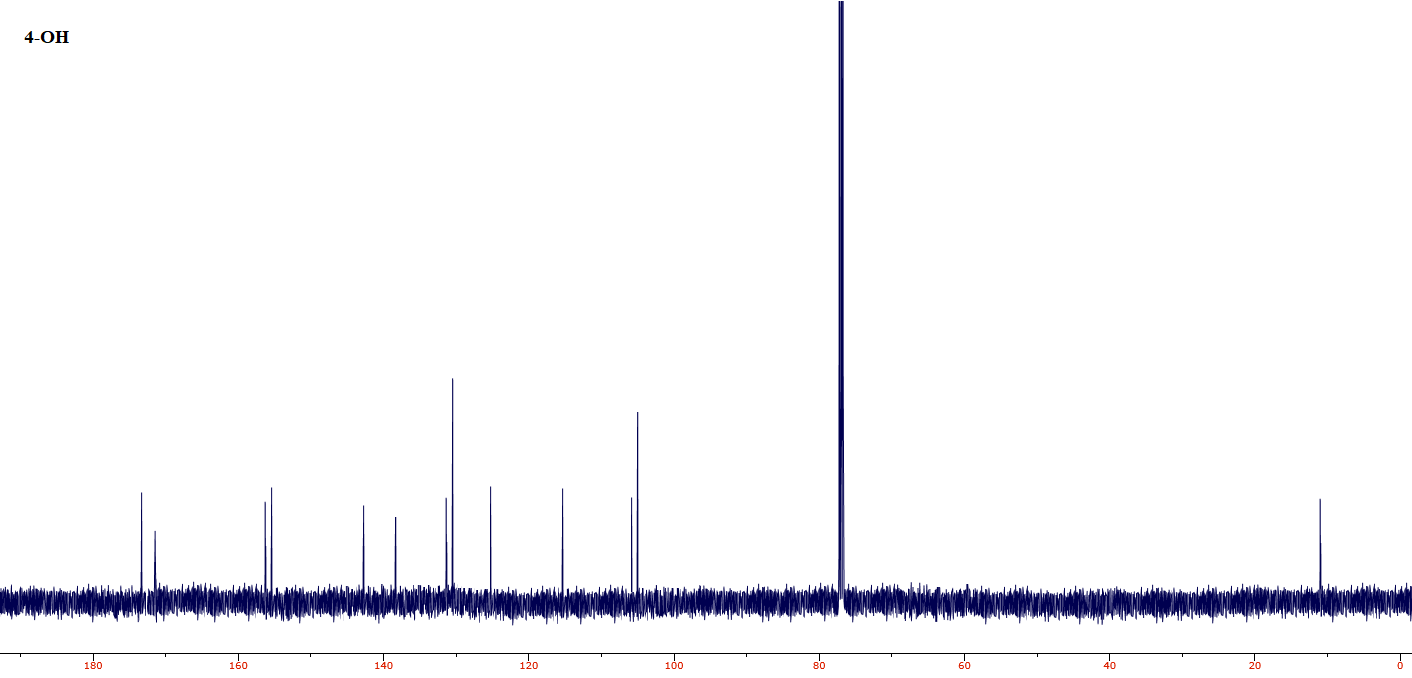
**

**-4 -**

**
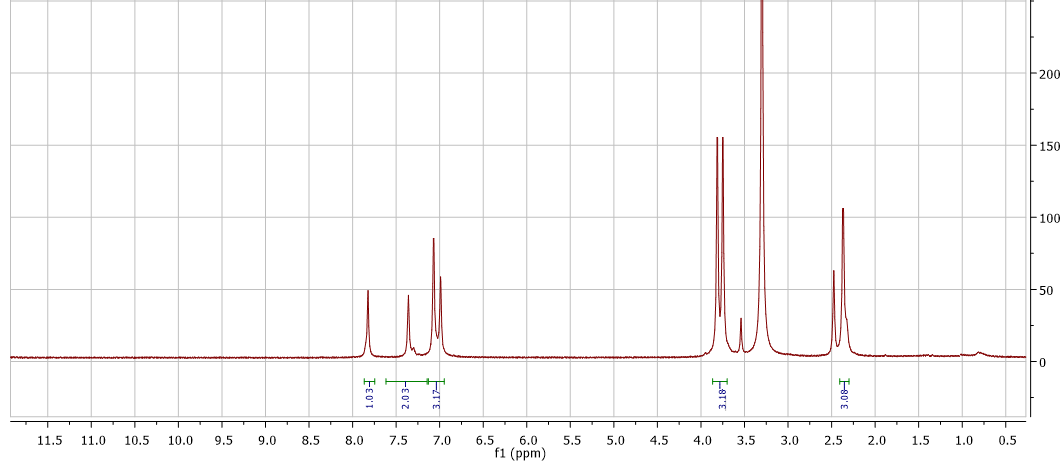
**

**
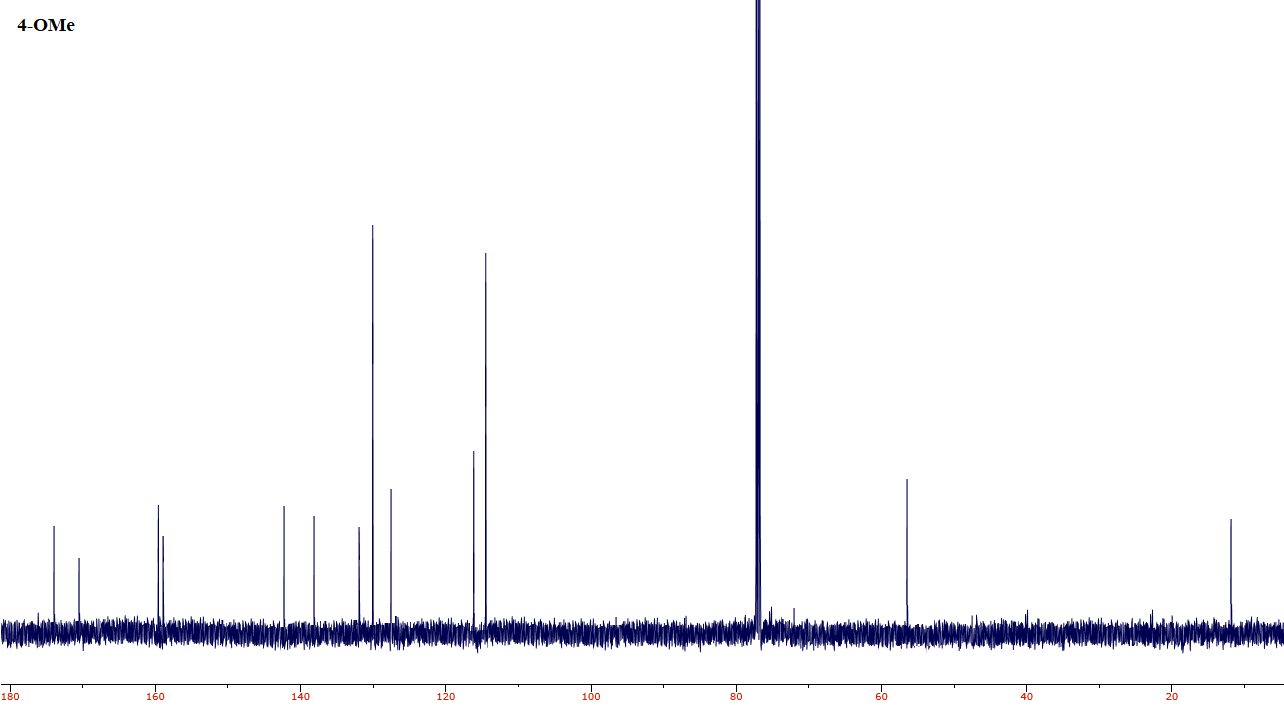
**

**-5-**

**
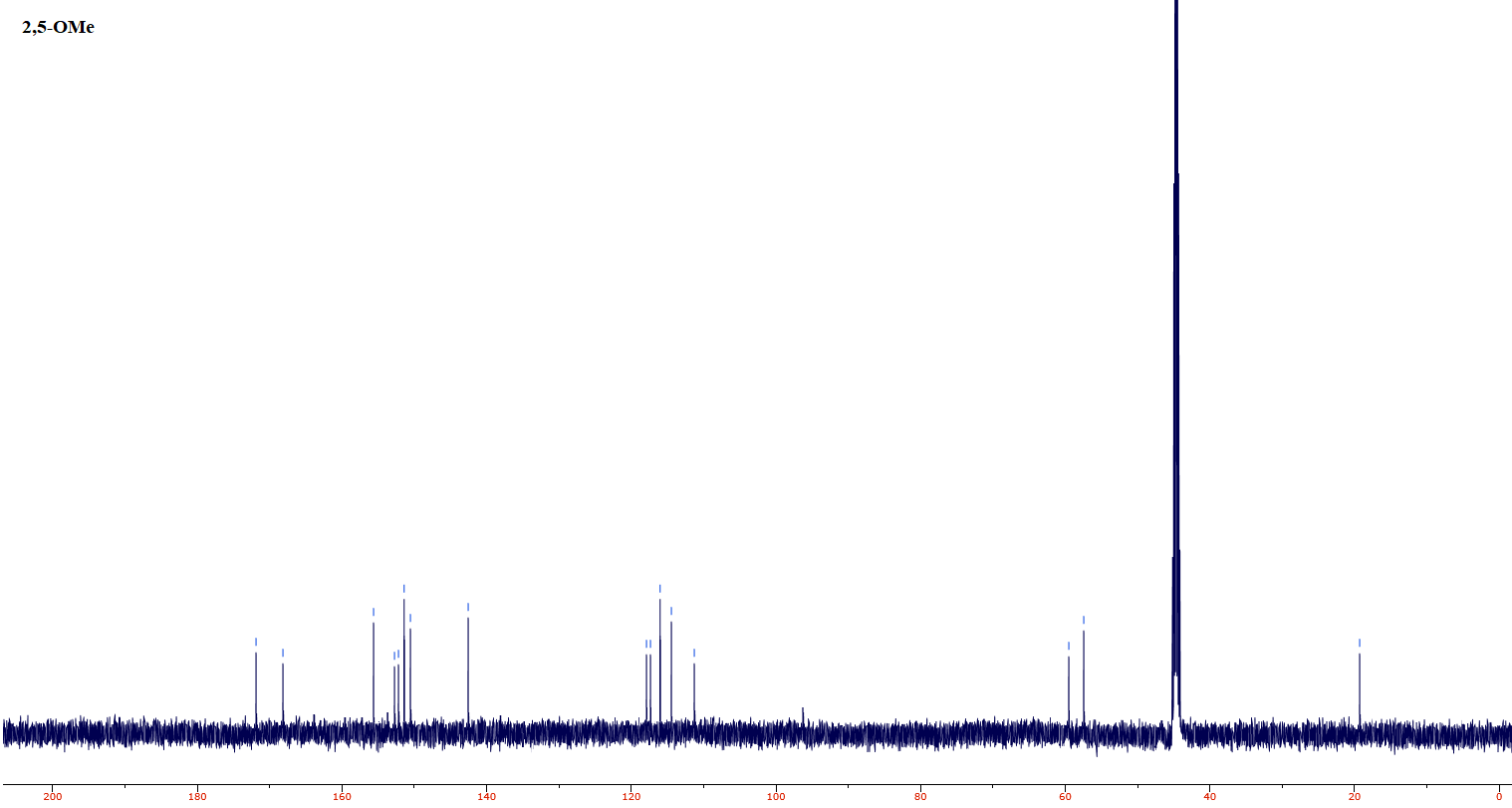
**

**-6-**

**
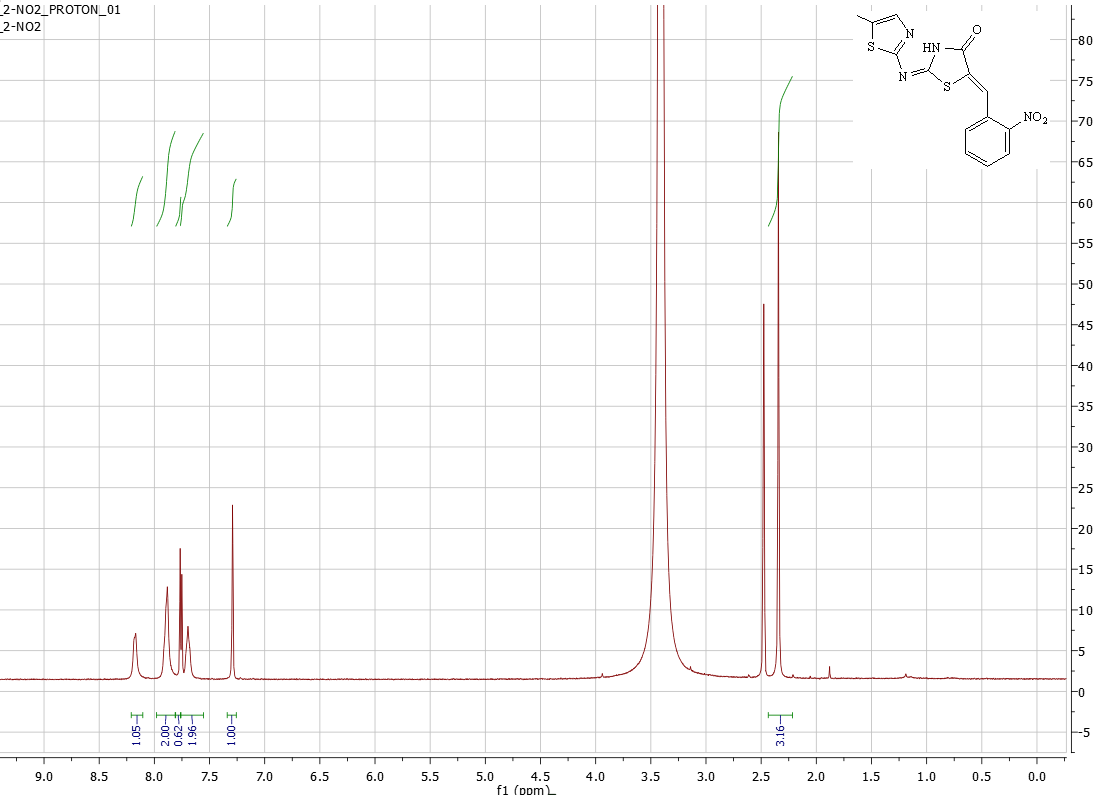
**

**
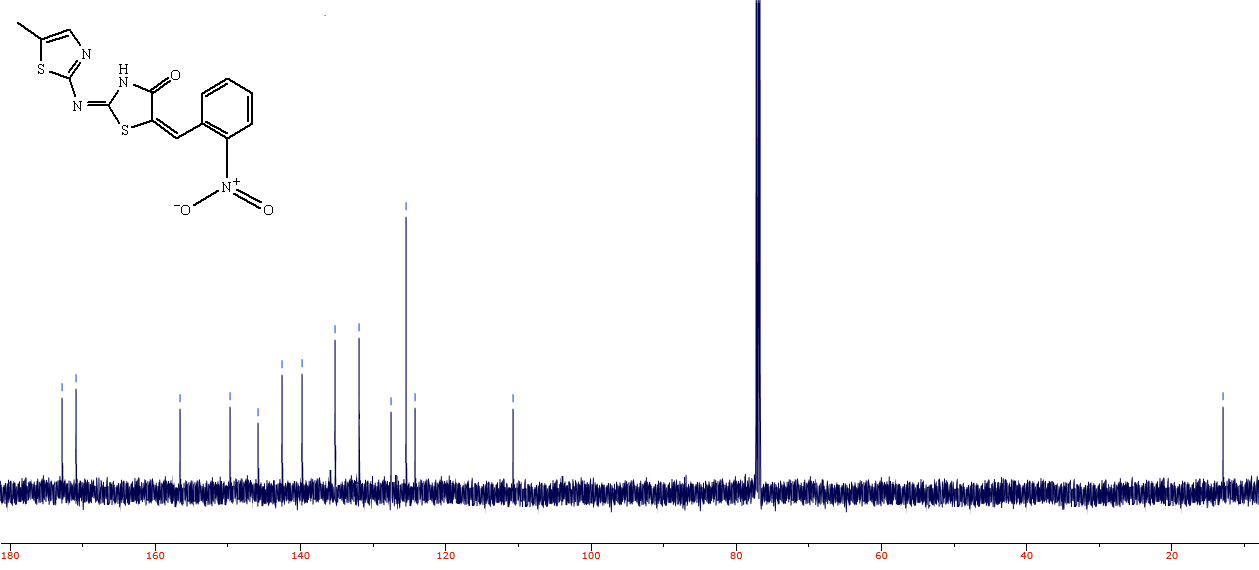
**

**-7-**

**
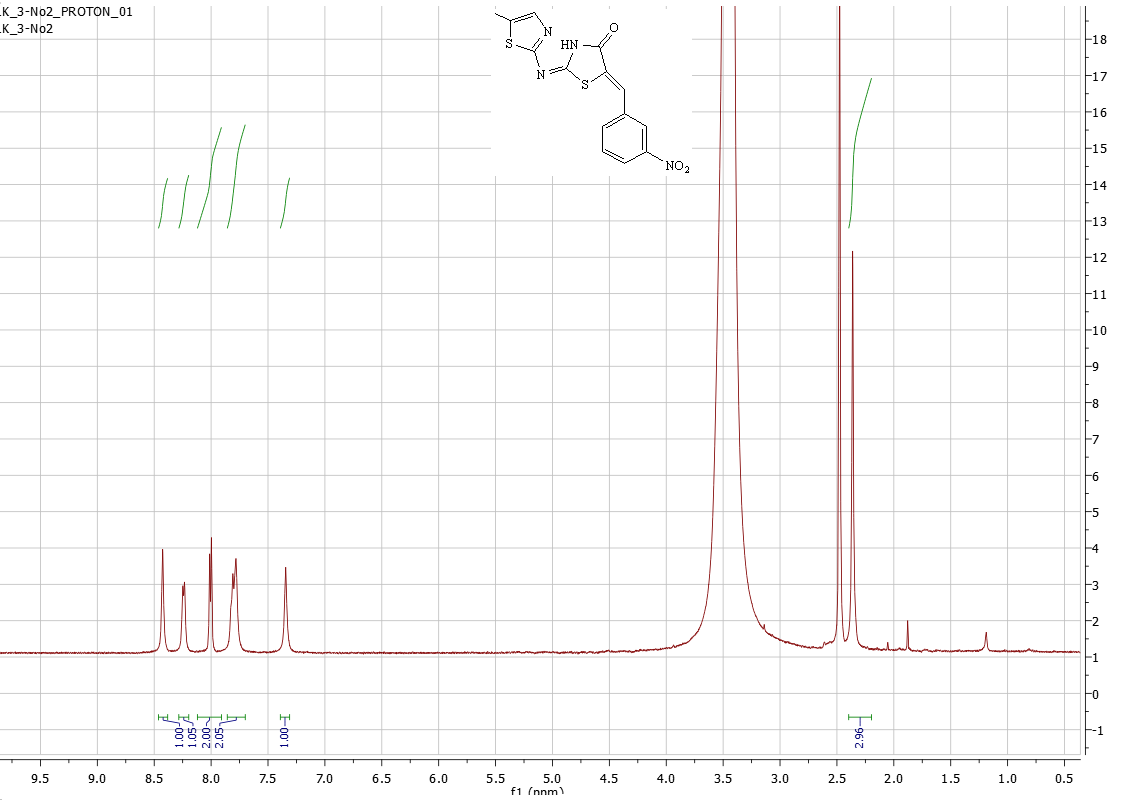
**

**
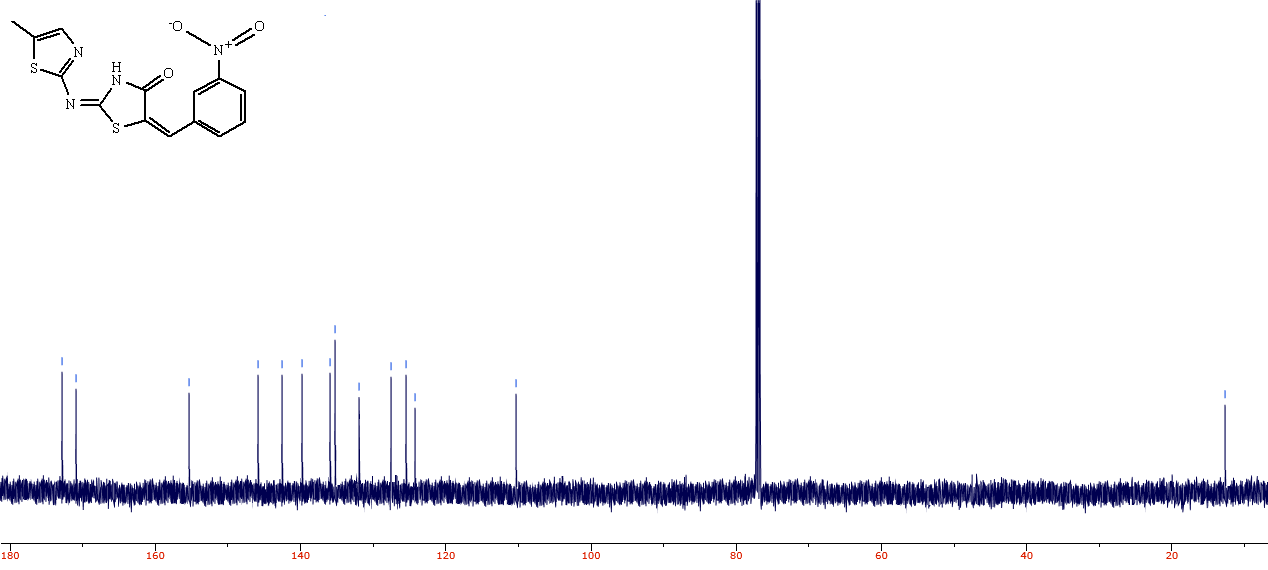
**

**-8-**

**
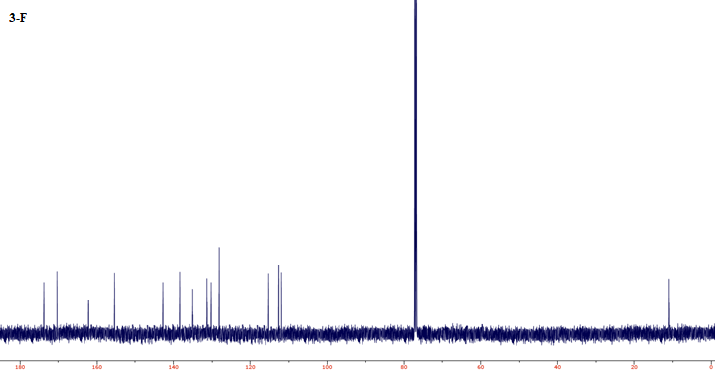
**

**-9-**

**
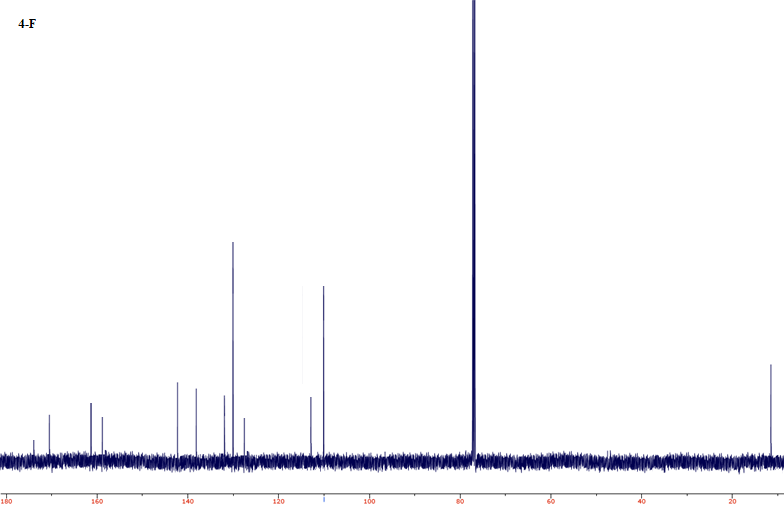
**

**-10-**

**
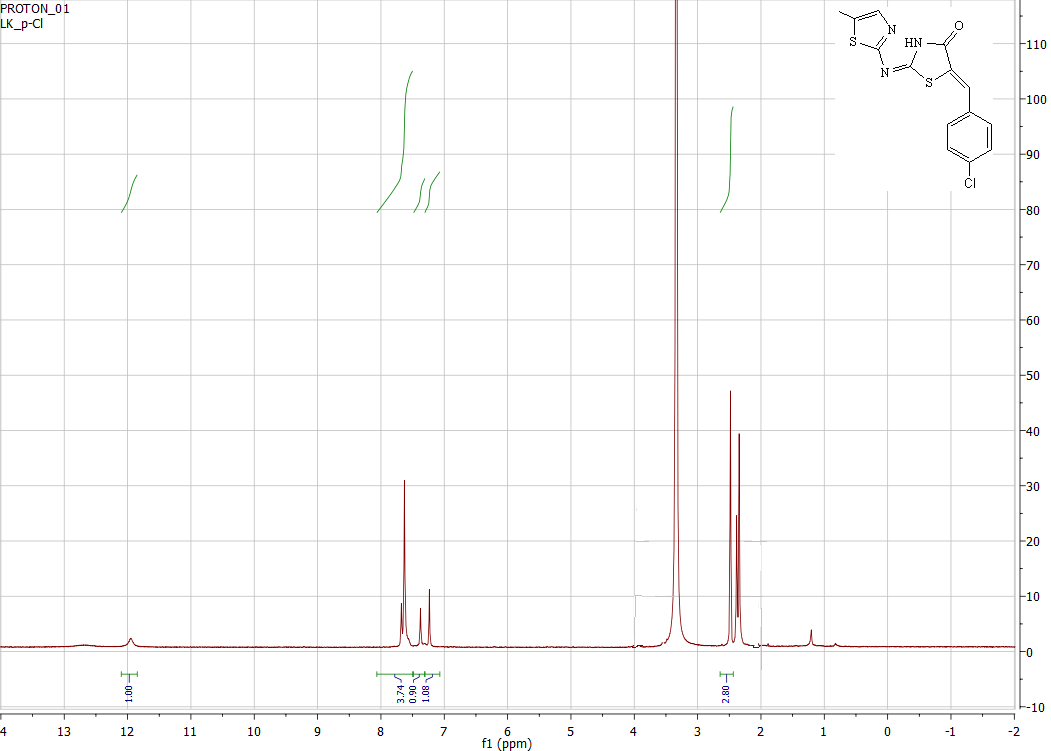
**

**
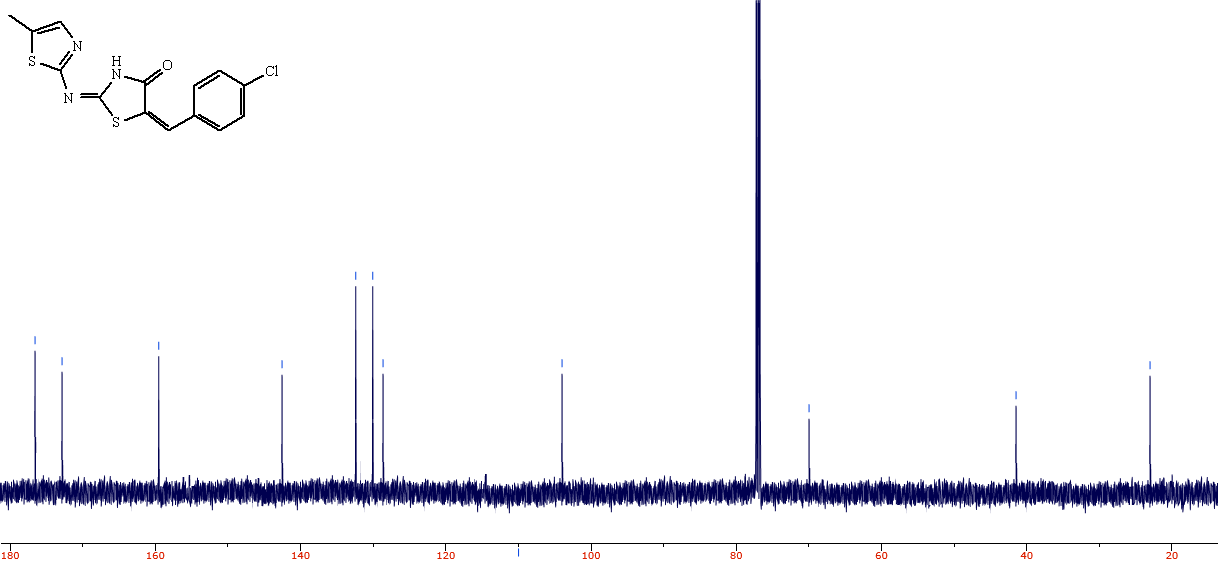
**

**-11-**

**
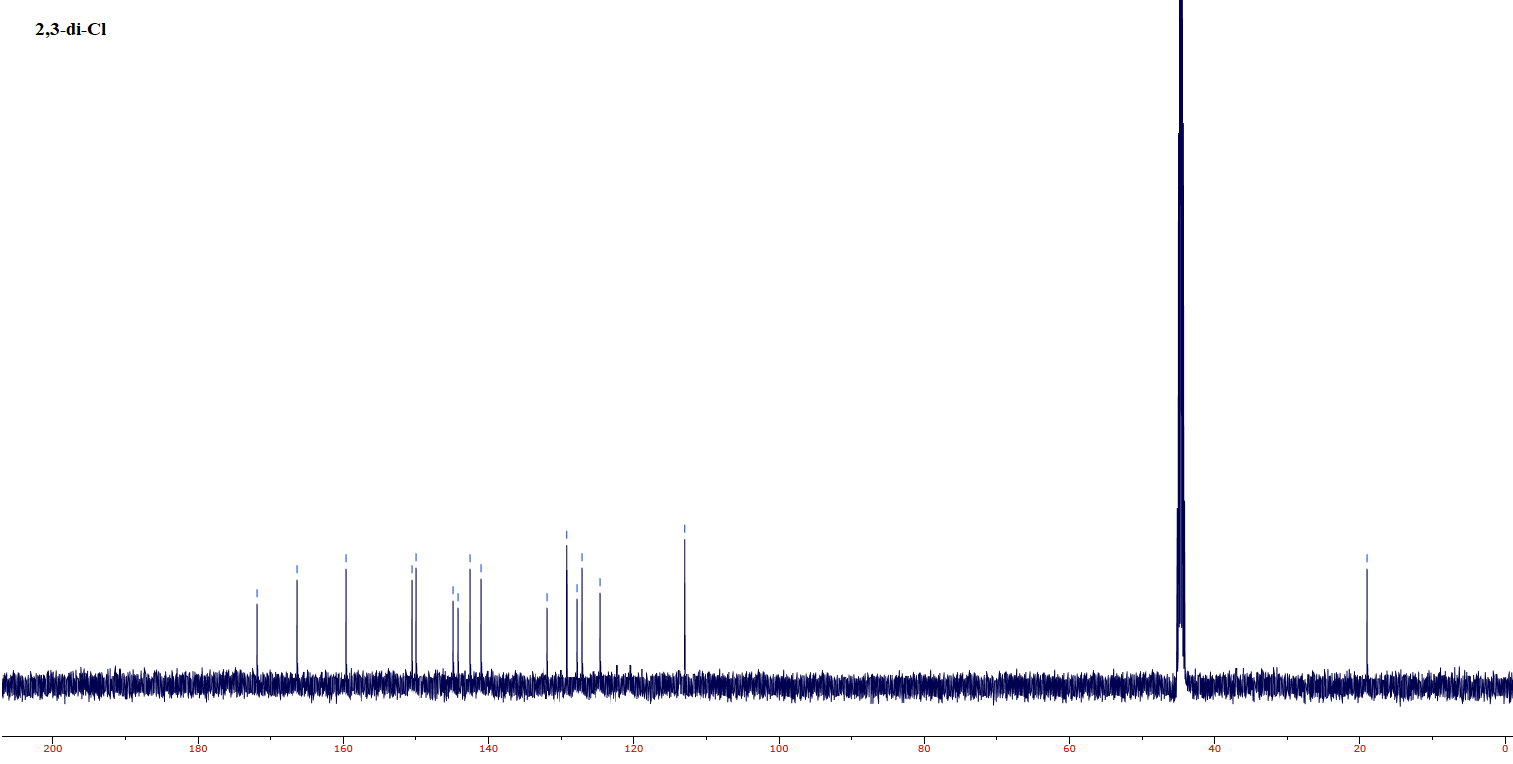
**

**-12-**

**
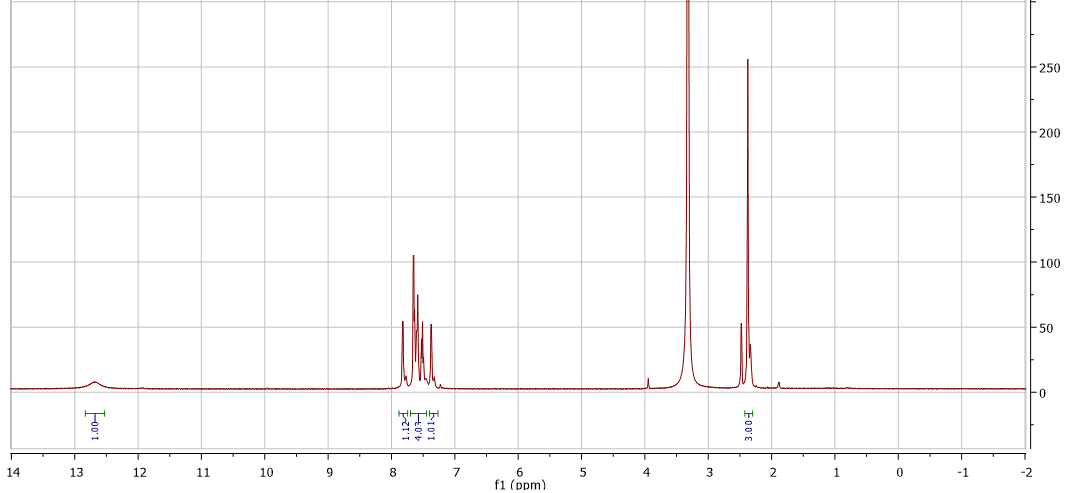
**

**
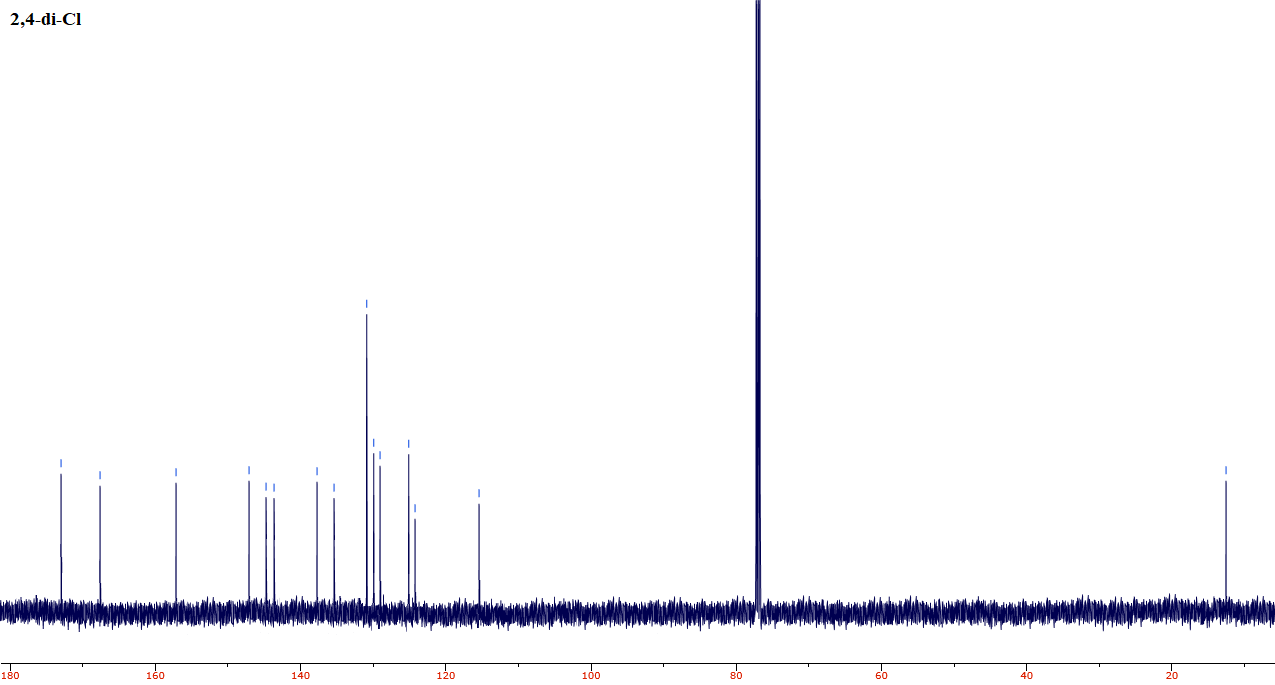
**

**-13-**

**
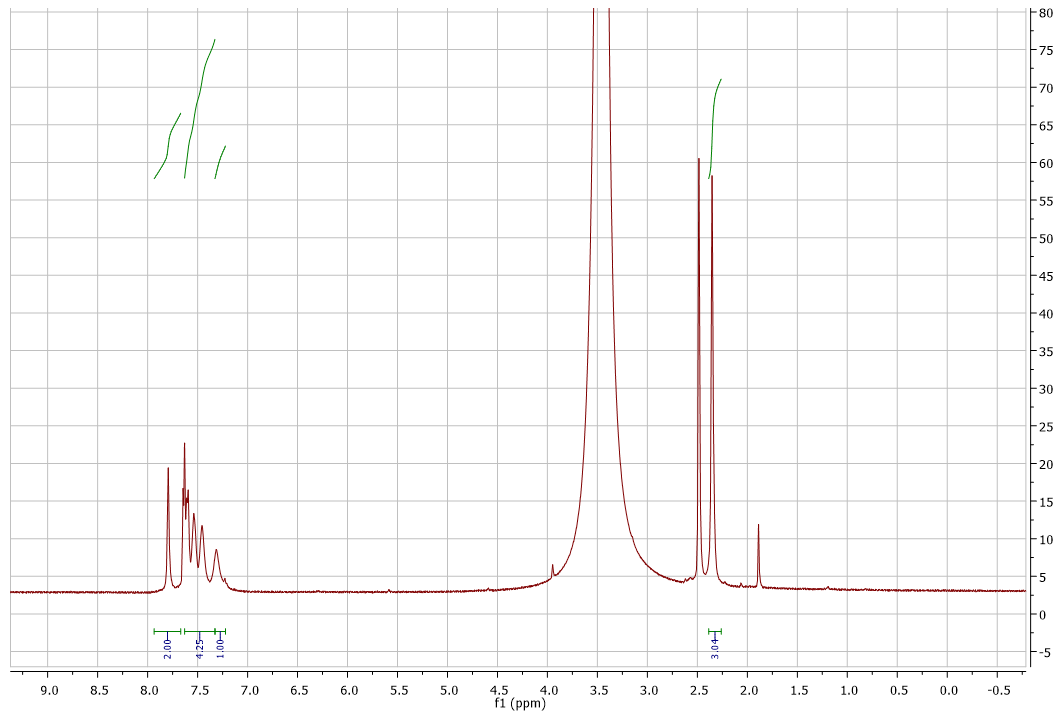
**

**
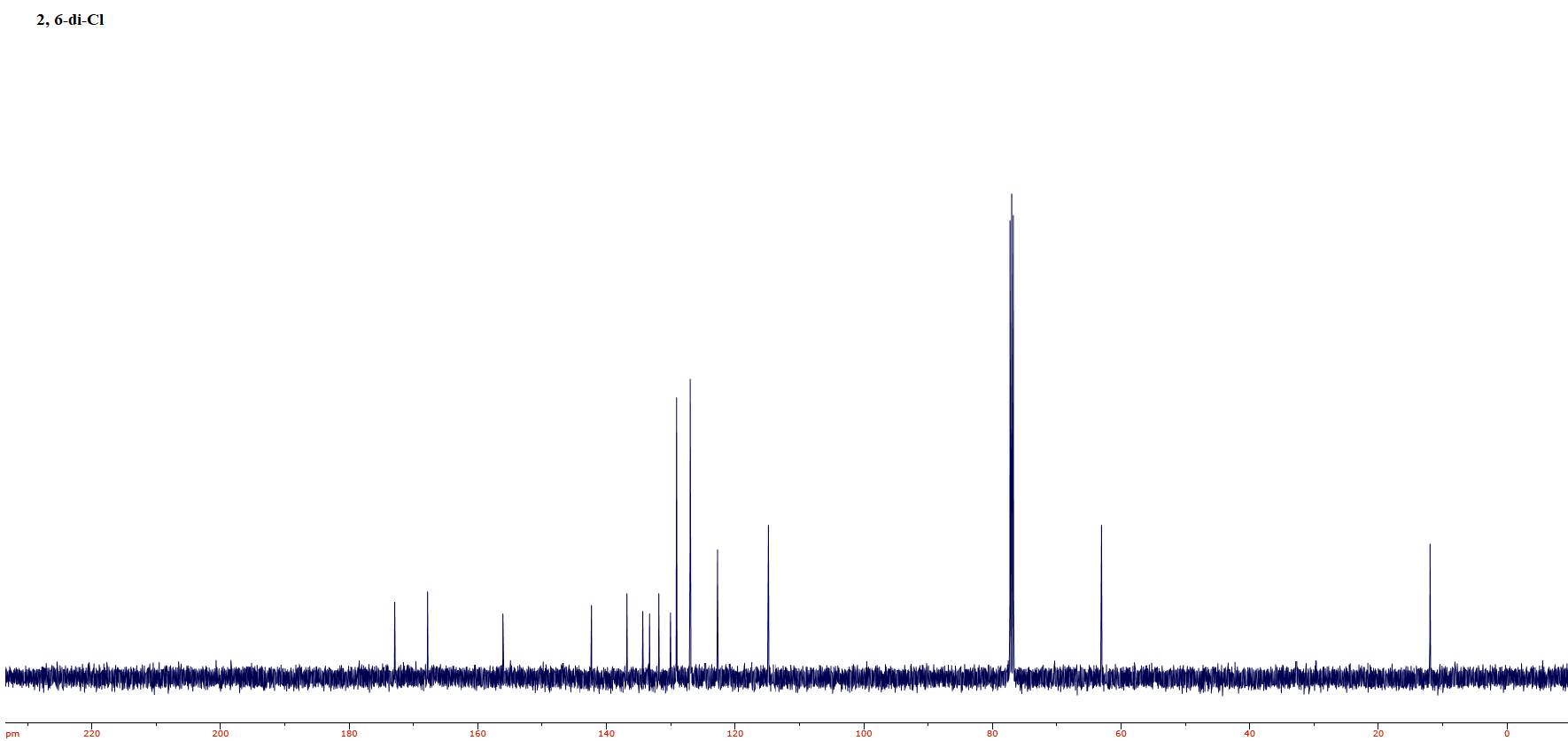
**

**-14-**

**
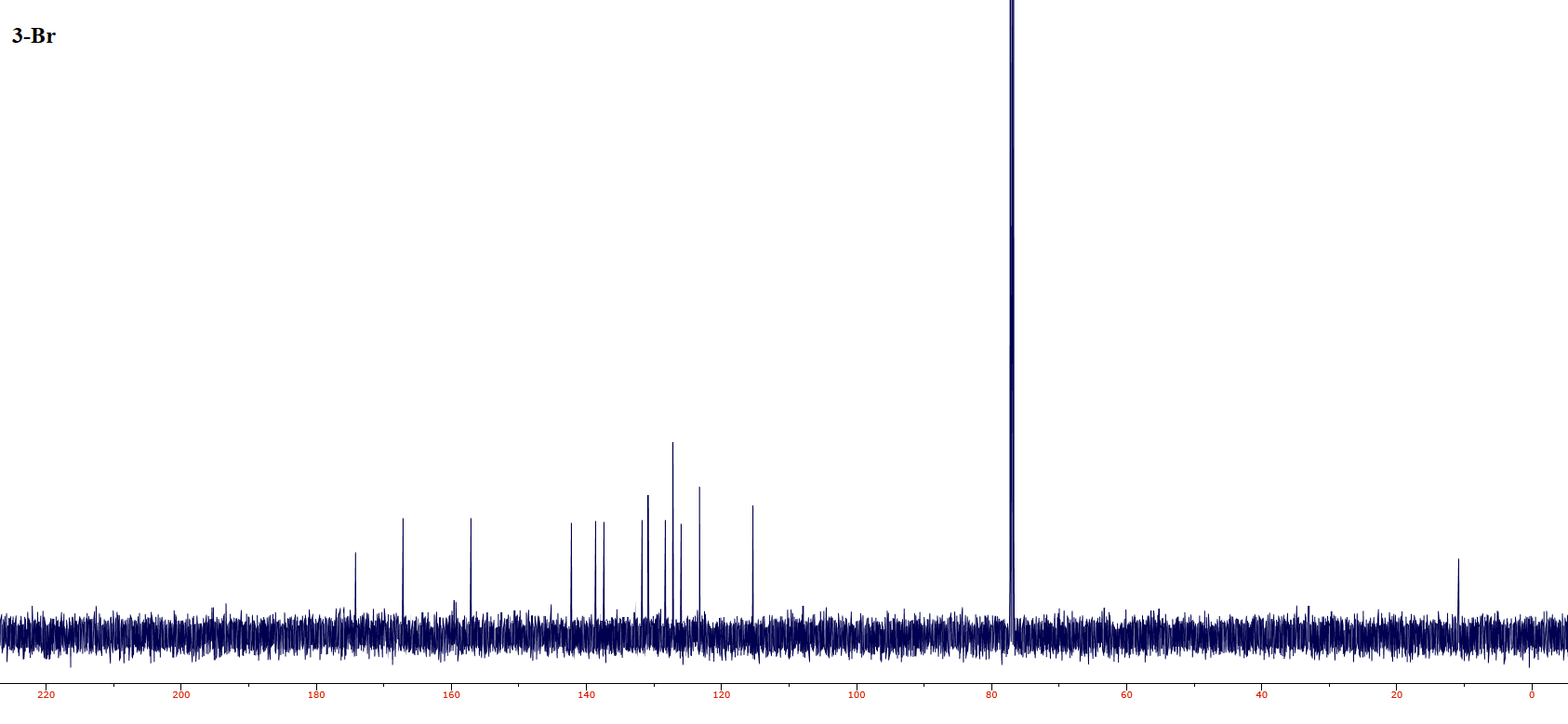
**

**-15-**

**
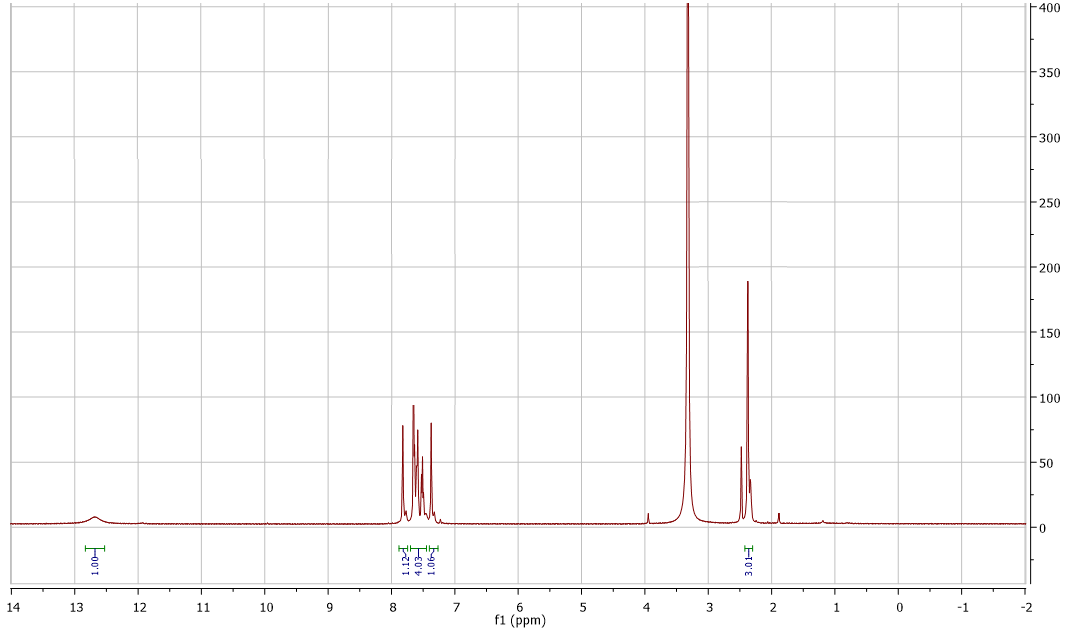
**

**
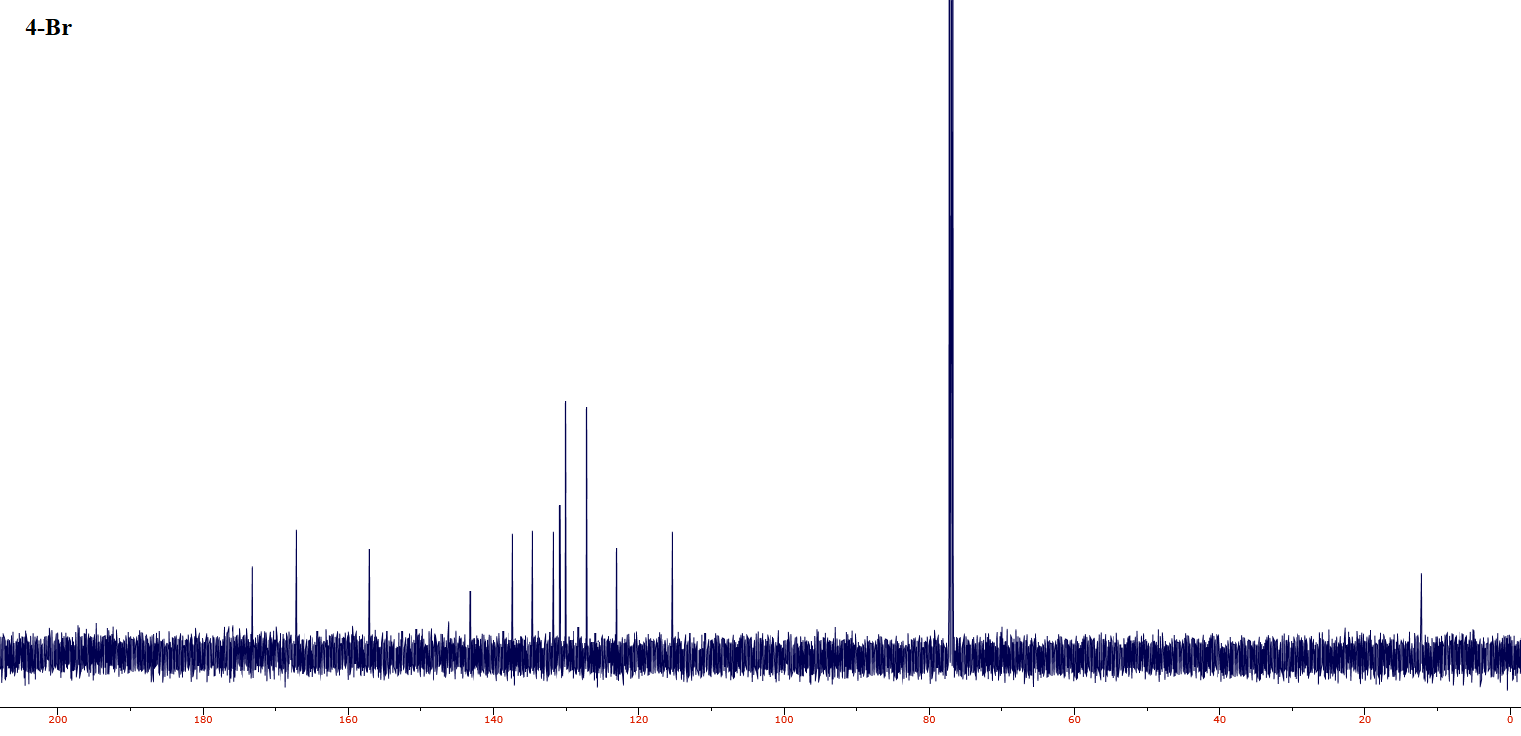
**

Supplement: Supplementary file 1 [file antibiotics-10-00309-s001.zip › antibiotics-1060612-supplementary/Supplementary files/1H-13C-NMR.docx]
